# Phenolic Compounds of *Justicia gendarussa* Show Pharmacological Potentials Against Pain, Oxidation, Hyperglycemia, Diarrhea, and Microbes: Phytopharmacological and Computational Approaches

**DOI:** 10.1155/bmri/2561508

**Published:** 2025-12-09

**Authors:** Farzana Akter Munny, Mehedi Islam, Mahafuza Akter, Mushtahsin Ferdousi, Md. Moaz Ahmed Asif, Md. Solaiman Hossain, Sabrina Sharmin, Md Zahidul Islam, Md. Aslam Hossain, Md. Rabiul Islam, Baisakhi Banerjee

**Affiliations:** ^1^ Department of Pharmaceutical Chemistry, Faculty of Pharmacy, University of Dhaka, Dhaka, Bangladesh, du.ac.bd; ^2^ School of Pharmacy, BRAC University, Dhaka, Bangladesh, bracu.ac.bd; ^3^ Department of Pharmaceutical Technology and Biopharmaceutics, School of Health and Life Sciences, North South University, Dhaka, Bangladesh, northsouth.edu; ^4^ Department of Pharmacy, Faculty of Science and Engineering, University of Information Technology and Sciences, Dhaka, Bangladesh; ^5^ Department of Pharmacy, School of Pharmaceutical Sciences, State University of Bangladesh, Dhaka, Bangladesh, sub.edu.bd; ^6^ Department of Biotechnology and Genetic Engineering, Jahangirnagar University, Dhaka, Bangladesh, juniv.edu

**Keywords:** analgesic, antidiarrheal, antioxidant, hypoglycemic, *Justicia gendarussa*, molecular docking, polyphenols

## Abstract

**Background:**

*Justicia gendarussa* is a branched shrub spread across Indian, Sri Lankan, and Malaysian forests. It has been widely used across many countries to treat asthma, rheumatism, colics in children, eczema, and HIV. The study goal was to investigate the phytoconstituents from *J. gendarussa* and to discover its therapeutic potential against various disease conditions.

**Methods:**

The plant sample was collected, dried, and grinded into coarse powder which was then soaked in methanol for 2 weeks. After the maceration process, the crude methanolic extract was subjected to solvent–solvent partitioning into four different fractions: n‐hexane soluble fraction (HSF), dichloromethane soluble fraction (DMSF), ethyl acetate soluble fraction (EASF), and aqueous fraction (AQF). DMSF was chemically evaluated through chromatographic separation, and all the fractions including the crude methanolic extracts were assessed for their potential pharmacological activities against pain, oxidative stress, hyperglycemia, diarrhea, and microbes following standard protocols.

**Results:**

Chemical investigation results in the isolation of lupeol, *β*‐sitosterol, and 1‐monostearin. The structures of the compounds were elucidated through meticulous NMR spectroscopic analysis. In a DPPH free radical scavenging assay, prominent action was noticed by EASF, with a median inhibition concentration (IC_50_) of 24.207 g/mL in comparison to the BHT with an IC_50_ value of 23.159 g/mL. In central analgesic activity, all the results were highly significant, with the highest (233.47%) time elongation in comparison to the control, observed after 90 min at 600 mg/kg b.w. and maximum peripheral analgesic activity of 61.96% was found at a dose of 600 mg/kg b.w. Two test doses (600 and 400 mg/kg b.w.) demonstrated substantial hypoglycemic and antidiarrheal effects that became more pronounced over time. The isolated compounds demonstrated impressive binding scores when interacting with glutathione reductase (3GRS), mu‐opioid receptor (MOR), kappa opioid receptor (KOR), and glucose transporter 3 (GLUT 3) receptors. However, their performance was notably lacking in terms of binding with cyclooxygenase‐2 (COX‐2) and dihydrofolate reductase (DHFR) receptors.

**Conclusion:**

Three isolated phytochemicals demonstrate promising binding affinities with the receptor molecules that support the pharmacological findings of this study. However, additional research needs to be conducted to isolate more phytoconstituents and affirm the pharmacological potential of *J. gendarussa*.

## 1. Introduction

Plant‐based medicines, derived from herbs, roots, flowers, and other botanical sources, are highly valued for their potential to treat a wide range of health conditions while often creating fewer side effects than synthetic pharmaceuticals. Increasing awareness of using natural remedies and a preference for holistic health approaches over lab‐made medicines were the driving force for a notable surge in the popularity of natural products and their utilization for curing pathological conditions [1]. In addition, natural products have a biological and molecular diversity that allows them to be used as unique templates for structural modifications as well as drug creation in the future, thus leading to the availability of safer and effective medicine [2]. Therefore, scientific research continues to uncover the biochemical mechanisms behind these plants′ therapeutic effects, validating their efficacy and safety.

*Justicia gendarussa*, a member of the Acanthaceae family within the Tracheophytes phylum and Angiosperms class, is a small shrub inhabiting tropical and subtropical regions. It exhibits a widespread distribution across Asian nations, including China, Indonesia, India, Sri Lanka, and Malaysia [3]. The plant is renowned for its rich chemical composition, encompassing alkaloids (e.g., justridisamide), flavonoids (e.g., quercetin, kaempferol, and naringenin), saponins (e.g., gendarussin), aromatic amines (e.g., 2‐amino benzyl alcohol and benzyl alcohol), fatty acids (e.g., oleic acid and 6,9,12‐octadecadienoic acid), and essential oils [4, 5]. These constituents contribute to its diverse biological activities.

The plant, widely recognized in traditional Chinese medicine, has historically been exploited for dealing with various ailments including injuries, rheumatic disorders, cardiac conditions, bacterial infections, sickle cell anemia, and inflammatory conditions such as muscular pain, respiratory issues, and bronchitis [6, 7]. Additionally, phytochemical investigations revealed phytoconstituents such as naringenin, kaempferol, and gendarussin A from the leaf extracts of *J. gendarussa* whereas apigenin was obtained from the root extract. These compounds were reported to possess anti‐inflammatory and cytotoxic properties [8–10]. Furthermore, justiprocumin A and justiprocumin B were isolated and characterized from the stem extract of *J. gendarussa* and found to have cytotoxic activity [11]. 6,8‐Di‐C‐*α*‐L‐arabinosyl‐apigenin, 6‐C‐*α*‐L‐arabinosyl‐8‐C‐*β*‐D‐xylosyl‐apigenin, and justidrusamides A‐D are obtained from the Indonesian *J. gendarussa* leaf extracts whereas *β*‐sitosterol, friedelin, and lupeol are reported in the Indian species of *J. gendarussa* [12]. Again, research further highlighted its multifaceted pharmacological properties including antioxidant, antifungal, hepatoprotective, antihelminthic, anticancer, anti‐HIV, antianxiety, larvicidal, and adulticidal activities [13, 14].

Phenolic compounds are known for their diversified biological activities. These properties are mostly attributed to their structural features which enable them to generate hydrogen bonds and hydrophobic interactions with the target protein molecules [15]. They show promising antioxidant effects by either inhibiting the formation of free radicals or scavenging them [16]. They have been shown to inhibit certain important enzymes such as *α*‐glucosidase or pancreatic lipase and thus provide the inhibitory responses [17].

Increased oxidative stress is the significant contributing factor to the development and progression of chronic, degenerative, and inflammatory conditions which result from an imbalance in redox homeostasis [18]. Reactive oxygen species (ROS) are pivotal to the pathogenesis of various conditions. The current pharmacological treatments for these disorders are often associated with adverse side effects, which can diminish patient compliance and compromise therapeutic outcomes. Antidiabetic medications, which are conventionally used for diabetic treatment, can assuage the severity of the disease but are associated with diverse adverse effects like hypoglycemic shock, edema, impaired liver and kidney function, or problems with the digestive or respiratory system [19]. Again, the conventional analgesic drugs also possess undesirable effects that necessitate the discovery of better compounds for their management [20]. The widespread consumption of antibiotics is generating antibiotic resistance, a worldwide health issue. Methicillin‐resistant *Staphylococcus aureus* (MRSA) alone is responsible for close to 50,000 deaths in the United States and Europe each year, and this scenario is much worse in undeveloped and developing countries because of the extent of its irrational use [21]. Again, the global prevalence of 2.5 billion diarrheal cases is reported by UNICEF and WHO, whereas 1.9 million children under 5 years of age die each year. Around 78% of child deaths in the developing countries of the Southeast Asian and African regions have mainly resulted from diarrhea [22]. Although there have been diverse treatment strategies, we might require therapeutic alternatives owing to their adverse effects. The plant kingdom offers a rich repository of natural compounds that can perform as therapeutic alternatives to conventional medications with greater efficiency and fewer adverse effects.

Phytochemical research has yielded innumerable secondary metabolites with diverse pharmacological properties. These bioactive secondary metabolites have been utilized as an origin of promising drug candidates and for developing new innovative therapies to treat major diseases. Although several studies have been conducted to explore anti‐inflammatory, cytotoxicity, antimicrobial, or antioxidant properties of *J. gendarussa*, there has been no report to our knowledge to investigate its antidiarrheal or hypoglycemic properties. Again, the phytochemical investigation on *J. gendarussa* is also limited. Thus, the purpose of this experiment was to assess the in vitro antimicrobial and antioxidant properties and in vivo analgesic, antidiarrheal, and hypoglycemic effects of *J. gendarussa*, as well as to conduct a phytochemical screening of the plant. Furthermore, in silico approaches were utilized to predict the pharmacological findings.

## 2. Materials and Methods

### 2.1. Sample Collection and Preparation

In January 2022, *J. gendarussa* leaves were acquired from Gazipur, Bangladesh. A Bangladesh National Herbarium (BNH) plant taxonomist accurately recognized the plant and created an accession number for future reference (DSCB‐65133). The leaves were thoroughly cleaned and meticulously chopped into small fragments and then shade‐dried in the open air for 7 days until they were completely dry. By using a high‐grade grinding machine, the dried pieces of leaves were pestled into a coarse powder, and a 1.15‐kg sample of the powdered material was collected for further investigation and analysis.

### 2.2. Instrumentations, Pharmaceuticals, and Chemicals

Nuclear magnetic resonance (NMR) spectra were acquired using a Bruker instrument at 400 MHz with deuterated chloroform (CDCl_3_) as the solvent. Solvent evaporation was performed using a Buchi Rotavapor made in Germany. Gel permeation chromatography (GPC) was accomplished utilizing Sephadex LH 20, and the compound analysis was conducted using Silica Gel 60 F 254 precoated thin‐layer chromatography (TLC) plates sourced from Merck, Germany. Using the vanillin/H_2_SO_4_ reagent, the spots on TLC plates were observed under UV light. The drugs utilized in the experiments, including diclofenac sodium (DS), ascorbic acid (ASA), glibenclamide, and loperamide, are acquired from Square Pharmaceuticals Ltd., a pharmaceutical company based in Bangladesh. All the reagents and chemicals, including acetic acid, 2,2‐diphenyl‐1‐picrylhydrazyl (DPPH), Folin–Ciocalteu reagent (FCR), gallic acid, Tween 80, and tert‐butyl‐1‐hydroxytoluene (BHT), were of analytical grade and taken from trustworthy manufacturers, including Active Fine Chemicals Ltd. (Bangladesh), DaeJung (Korea), and Merck (Germany).

### 2.3. Test Microorganism

Gram‐positive bacteria include *Bacillus subtilis (ATCC 6051)*, *Bacillus megaterium (ATCC 14581)*, *Bacillus cereus (ATCC 14579)*, *Staphylococcus aureus (ATCC 25923)*, and *Sarcina lutea (ATCC 4698)*. Gram‐negative bacteria include *Salmonella paratyphi (ATCC 9150)*, *Pseudomonas aeruginosa (ATCC 27853)*, *Shigella boydii (ATCC 9207)*, *Escherichia coli (ATCC 11775)*, *Shigella dysenteriae (ATCC 13313)*, *Vibrio mimicus (ATCC 33653)*, *Salmonella typhi (ATCC 6539)*, *and Pseudomonas anguilliseptica (ATCC 19320)*. The experimental fungi include *Candida albicans (ATCC 10231)*, *Saccharomyces cerevisiae (ATCC 9763)*, and *Aspergillus niger (ATCC 16404)*. These microorganisms were used for the antimicrobial assay and obtained as pure cultures from the Ben Medical Research Center (BRC), University of Dhaka.

### 2.4. Experimental Approach

#### 2.4.1. Plant Material Extraction

To extract bioactive compounds from *J. gendarussa*, 1.15 kg of air‐dried plant material was soaked in 97% methanol for 14 days. Throughout this period, the container was periodically swirled and shaken to ensure complete extraction. After the 2‐week cold extraction period, the mixture was refined and processed utilizing a Buchi Rotavapor. A large funnel fitted with a cotton plug was used to aid the filtration process. The cold extraction process was repeated several times over 6 days, with the resulting dried extracts accumulated in the same beaker. The final dried methanol extract from the entire process weighed 37 g, representing 3% of the initial material weight.

#### 2.4.2. Partitioning for Biological Screening

The Kupchan method was utilized for solvent–solvent partitioning [23]. A crude methanol extract was initially partitioned into separate fractions using the modified Kupchan method. The process yielded four main fractions: HSF (1.5 g), DMSF (1.4 g), EASF (1.6 g), and AQF (0.7 g). Each fraction was individually evaporated utilizing a rotary evaporator.

#### 2.4.3. Compound Isolation

DMSF of *J. gendarussa was* fractionated by applying GPC Lipophilic Sephadex LH 20. The use of a gradient solvent solution as the mobile phase permits molecular separation based on size and polarity. Sixty test tubes were collected after GPC, and each test tube held approximately 3 mL of liquid. These fractions were then screened using commercially available TLC plates, which are aluminum‐backed plates with dimensions of 20 × 20 cm, precoated using silica gel (Kieselgel 60 PF254). The plates were formed in a solvent system, and the separate compounds were then visualized using UV light and reagent spray. Then, the bands were scraped and eluted with the appropriate solvent before being dried at room temperature to yield compound crystals.

#### 2.4.4. Structural Characterization of the Isolated Compounds

The ^1^H NMR spectra in CDCl_3_ of the three separated compounds were recorded using a Bruker spectrometer of frequency 400 MHz, with the *δ* values in relation to the remaining nondeuterated solvent signal. Relative to the solvent peak, chemical shifts produced by each compound were recorded in ppm units and coupling constants were recorded in Hz.

#### 2.4.5. Experimental Animals

Swiss albino mice (4–5 weeks old, both male and female), each weighing between 20 and 25 g, sourced from the Animal Resource Branch of the International Centre for Diarrheal Diseases and Research, Bangladesh (ICDDR, B), served as animal models in the study. The mice had unlimited access to food and water in their plastic cages. The parameters of the animal home were kept constant, including a photoperiod of 12 h of natural light per day, relative humidity of 55%–60% at a temperature of 28°C–31°C, and the relative humidity was between 55% and 60%. Because mice are highly sensitive to changes in their surroundings, they were housed in the laboratory for 7 days to acclimate. Throughout the experimentation, standard protocols for the supervision and use of laboratory animals were strictly observed. The protocol and all ethical aspects were thoroughly reviewed and approved by the Animal Ethics Committee of the State University of Bangladesh (Ref. 2025‐01‐05/SUB/I‐ERC/001).

Twenty mice were utilized in total for this study. There are five rodent groups, each with four rats in them. While the negative control group had been administered 1% Tween 80 dissolved in normal saline (10 mL/kg), the positive control group was given the usual medicine. There were five separate groups for every test, which were as follows:

• Group I = negative control group (CTL)
• Group II = positive control group (STD)
• Test Group I = *J. gendarussa* methanolic crude extract 200 mg/kg (JGME 1)
• Test Group II = *J. gendarussa* methanolic crude extract 400 mg/kg (JGME 2)
• Test Group III = *J. gendarussa* methanolic crude extract 600 mg/kg (JGME 3)

The same mice were utilized for the in vivo studies and a resting phase of 7 days was given to the mice between the studies.

### 2.5. Antioxidant Assay

#### 2.5.1. DPPH Free Radical Scavenging Assay

The plant extract is diluted several times to determine its antioxidant activity. A solution of DPPH, a stable free radical, is combined with 3 mL of each dilution, containing 2 mL each. The DPPH usually has a purple color, and if the plant extract has antioxidants, the DPPH radical will receive a hydrogen atom from the extract, turning it into a colorless substance. The plant extract′s antioxidant activity increases with the degree of decolorization.

A recognized antioxidant, for example, BHT, is compared to an estimate of the antioxidant potential of the plant extractives. The absorbance of the solution is determined at 517 nm by utilizing a UV‐Vis spectrophotometer. Lower levels of antioxidant activity will be indicated by a higher absorbance value or less decolorization [24]:

% Inhibition=Ablank−AsampleAblank×100%

#### 2.5.2. Total Phenolic Content Determination

The reference standard and the oxidizing agent used to quantify the total phenolic content of the extracts were gallic acid and FCR, respectively. We adhered to the Viticulture and Harbertson protocol. First, we combined 0.5 mL of the extract solution (2 mg/mL concentration) with 2.5 mL of FCR and 2 mL of sodium carbonate (Na_2_CO_3_). After that, we let the mixture sit at room temperature for 20 min. Using a UV spectrophotometer, we determined the solution′s absorbance at 760 nm after it had been incubated. Next, using various amounts of gallic acid, we produced a standard curve. We determined the extract′s total phenol concentration by comparing the extract solution′s absorbance to the standard curve. The amount of phenolic content was figured in mg of gallic acid equivalent (GAE) per gram of extract [24].

### 2.6. Analgesic Assay

#### 2.6.1. Central Analgesic Assay

The central analgesic potential of the crude extract was assessed by the tail immersion test [25]. DS was utilized as the positive control. Before the experiment began, the experimental animals were fasted for a duration of 18 h. The negative control group was administered 10 mL/kg of a 1% Tween 80 in typical saline solution. In contrast, the reference group received a 5 mg/kg oral solution of DS prepared in regular saline. The methanolic crude extract was administered to the three test groups, JGME 1, JGME 2, and JGME 3, at doses of 200, 400, and 600 mg/kg of b.w., respectively. Here, 1–2 cm of the mice′s tails was immersed in heated water with a constant temperature of 55°C. The reaction time is ascertained as the duration required for the mice to withdraw their tails from the water bath. This tail immersion time is estimated after 30, 60, and 90 min of oral administration of test samples and control. Comparing with the control, the % of time elongation was estimated, and it was estimated by applying the following formula:

% time elongation=average tail flicking time of test samples–average tail flicking time of the control groupaverage tail flicking time of the control group×100%

Central analgesic activity was evaluated using DS as a standard. A higher % of time elongation designates more significant central analgesic activity.

#### 2.6.2. Peripheral Analgesic Assay

The methanolic extract′s efficiency as a peripheral analgesic was also assessed using the acetic acid–induced writhing method [25]. Here, the doses for the negative control as well as treatment groups were administered as described in Section 2.6.1, while oral DS was administered at 5 mg/kg b.w. to the positive control group. After 30 min of their administration, 1% acetic acid was introduced into the experimental animals′ peritoneum (at 0.1 mL/10 g b.w.) to elicit pain perception. Each mouse in each group was monitored individually, starting 5 min after the introduction of the acetic acid solution intraperitoneally and the number of writhing episodes in 10 min was counted. The mice would sometimes initiate writhing but would not end it. Half‐writhing was the word used to denote this incomplete writhing, and two half‐writhings were counted as one whole writhing. The average writhing number was calculated for each group of mice. The % of inhibition of writhing was assessed in parallel to the control:

% inhibition of writhing=Ncontrol−NtestNcontrol×100%

The greater the peripheral analgesic activity, the higher will be the inhibition of writhing of that group. Thereafter, the test samples′ peripheral analgesic activity was compared to DS.

### 2.7. Antidiarrheal Assay

Shoba and Thomas′ castor oil–induced diarrhea method was used to evaluate *J. gendarussa* extract′s antidiarrheal activity [26]. Fifty‐milligram/kilogram b.w. loperamide was administered orally to the positive control group, while the negative control and treatment groups obtained their individual doses as outlined in Section 2.6.1. After 1 h of that, purified castor oil (1 mL) was introduced to every mouse to incite diarrhea. Separate cages were assigned to each rodent, with the help of blotting paper on the floor.

After administering castor oil, the diarrheal feces were enumerated for every mouse up to 4 h, counting at the end of every hour. Fresh alternate sheets of blotting paper were placed at the commencement of each hour. The antidiarrheal efficacy of *J. gendarussa* was examined in parallel with that of the control groups. By utilizing the equation shown below, the % diarrhea reduction was determined, and the antidiarrheal efficacy was assessed:

% reduction of diarrhea=P−QP×100%

where *P* and *Q* are the average diarrheal feces in the negative control and test groups, respectively.

### 2.8. Hypoglycemic Assay

An oral glucose tolerance test was conducted in the mice to assess the hypoglycemic efficacy of *J. gendarussa* [26]. Before the experiment was conducted, all rodents were fasted for 18 h. A one‐touch glucometer (Bioland G‐423S) to assess the blood glucose level of individual mice was employed at 0 h. The dose for the treatment groups and the negative control was administered in accordance with the instructions provided in Section 2.6.1. The positive control group was delivered with 10‐mg glibenclamide/kg of b.w. After waiting for 1 h, all groups were given 2 mg/kg b.w. of a 10% glucose solution orally. To assess the test samples′ hypoglycemic activity, blood samples were drawn from each mouse′s tail vein at 1, 2, and 3 h after glucose loading. The hypoglycemic effect was quantified by estimating the percentage of blood glucose levels reduced. The subsequent formula was employed to compute the percentage decrease in blood glucose levels:

% reduction in blood glucose level=P−QP×100%

where *P* and *Q* are the average blood glucose levels of the negative control and positive control or test groups, respectively.

### 2.9. Antimicrobial Assay

#### 2.9.1. Disc Diffusion Test

The antibacterial properties of various crude extract fractions were analyzed utilizing the disc diffusion method [24]. The test samples were evenly dispersed on sterilized filter paper discs (6 mm in diameter) and uniformly distributed in the nutrient agar medium seeded with the test microorganisms. Blank discs were used as negative standards, whereas typical kanamycin antibiotic discs on the market were deemed positive standards. These plates containing the samples were kept upside down for around 24 h at 4°C to naturalize the most efficient diffusion into the medium. The plates were then turned and incubated at 37°C for a duration of 24 h. A clean, distinct zone of inhibition was produced when the antibiotic samples penetrated deeply into the medium and stopped the growth of germs. To determine how effective the test sample was against bacteria, we measured the widths of the inhibition zones in millimeters.

### 2.10. Molecular Docking Study

The study used computational modeling to predict the receptor binding profiles of three detached chemical compounds from *J. gendarussa* and picked standard drugs opposed to their target proteins. To conduct the in silico study of three separated compounds of *J. gendarussa*, PyRx, PyMOL 2.3, and BIOVIA Discovery Studio Version 4.5 were used according to the semiflexible processes explored in numerous areas of research [27, 28].

#### 2.10.1. Target Protein Selection

The isolated compounds′ prospective bioactivities, such as antioxidant, analgesic, antidiarrheal, hypoglycemic, and antimicrobial activities, were identified through computational docking. The antioxidant, analgesic, hypoglycemic, antidiarrheal, and antibacterial characteristics are determined through the molecular interaction; glutathione reductase (PDB ID: 3GRS), kappa opioid receptor (PDB ID: 6VI4), cyclooxygenase‐2 (COX‐2) (PDB ID: 1CX2), glucose transporter 3 (GLUT 3) (PDB ID: 4ZWB), mu‐opioid receptor (PDB ID: 5C1M), and dihydrofolate reductase (DHFR) (PDB ID: 4M6J) proteins were chosen based on biochemical processes and existing research [27, 29–31]. The 3D crystal structures of the targeted proteins were accessed from the RCSB Protein Data Bank (https://www.rcsb.org; retrieved on August 8, 2024) in PDB format. The PyMOL 2.3 program was utilized to open each biomolecule collected and clear the protein of water molecules and undesirable residues.

#### 2.10.2. Ligand Preparation

**Figure 1:**
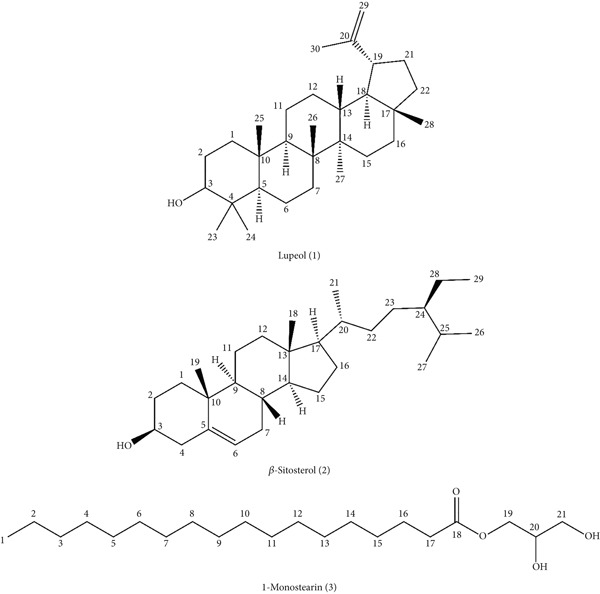
Structures of isolated phytochemicals from *Justicia gendarussa* using NMR technologies.

The structures of each detached chemical compound (1–3) are illustrated in Figure 1. These three compounds, for example, lupeol (PubChem CID: 259846), *β*‐sitosterol (PubChem CID: 222284), and 1‐monostearin (PubChem CID: 24699), were investigated through the PubChem database (https://pubchem.ncbi.nlm.nih.gov/; accessed on 8 August 2024). The three‐dimensional conformations of the ligands along with the standard drugs, for example, diclofenac (PubChem CID: 3033), BHT (PubChem CID: 31404), morphine (PubChem CID: 5288826), ciprofloxacin (PubChem CID: 2764), loperamide (PubChem CID: 3955), and glibenclamide (PubChem CID: 3488), were collected and stored in SDF format. The ligands were imported into Discovery Studio Version 4.5, and a ligand library was originated by applying their PubChem CIDs in PDB format. The PM6 semiempirical approach was employed to optimize both standard and isolated ligands to improve the precision interaction at the molecular level [28, 32].

#### 2.10.3. Ligand and Protein Interaction

For predicting the binding patterns and affinities of individual compounds with respect to macromolecules, molecular docking was implemented in this study [32]. The computational‐aided interaction method was performed using PyRx AutoDock Vina, a sophisticated software, employing a semiflexible modeling approach. The coveted protein was interjected and consigned as a macromolecule. An investigation was performed to ascertain the site‐specific ligand–protein interaction utilizing literature‐derived amino acids with three‐letter identifiers. The PyRx software was used to minimize energy by importing hybrid 3D conformers of ligands in SDF format. The PyRx AutoDock Vina program and Open Babel tool were applied to transform the ligands into pdbqt format to equip the best possible hit. Table 1 illustrates that the center of the grid box is the location of all active binding sites of the receptors. Throughout the docking procedure, the default configuration of all the other parameters was kept unchanged. AutoDock Vina (Version 1.1.2) was used to perform the docking process, and the results were extrapolated. Output files of macromolecules and ligands were collected in pdbqt format and combined to export PDB format for further visualization. The generation of both 2D and 3D figures was done utilizing the Discovery Studio Visualizer (Version 4.5).

**Table 1:** Selecting the site of the target and the target receptors′ grid mapping.

**Receptor**	**Standard**	**Target binding sites**	**Reference**	**Grid box**		
3GRS	BHT	VAL (102), LYS (127), ASN (129), VAL (130), GLN (131), LYS (143), SER (145), SER (147), GLY (148), ASP (183), and THR (185)	[28]	Center	X	51.3686
					Y	46.8836
					Z	18.7120
				Dimensions	X	35.3410
					Y	26.5713
					Z	25.0000

5C1M	Morphine	HIS 54, SER 55, GLN 124, ASN 127, TRP 133, ASP 147, TYR 148, MET 151, LEU 232, LYS 233, VAL 236, PHE 237, TRP 293, ILE 296, HIS 297, VAL 300, TRP 318, ILE 322, and TYR 326	[30, 31]	Center	X	−0.4432
					Y	14.1316
					Z	−47.6285
				Dimension	X	17.1067
					Y	17.6578
					Z	25.0000

1CX2	Diclofenac	LEU 15, ASN 34, CYS 36, ASN 39, CYS 41, GLN 42, ASN 43, ARG 44, GLY 45, GLU 46, CYS 47, GLY 135, PRO 153, ALA 156, GLN 461 GLU 465, LYS 468, and ARG 469	[30, 31]	Center	X	23.3530
					Y	20.8733
					Z	9.2931
				Dimension	X	21.0975
					Y	17.6384
					Z	25.0000

KOR	Loperamide	LEU 103, LEU 107, SER 136, ILE 137, TRY 140, ILE 180, TRP 183, LEU 184, SER 187, ILE 191, LEU 192 ILE 194, and VAL 195	[29]	Center	X	54.4689
					Y	−50.401
					Z	−15.8278
				Dimension	X	14.1579
					Y	26.9978
					Z	25.0000

GLUT 3	Glibenclamide	TYR 26, THR 28, GLY 29, VAL 30, LEU 167, THR 191, PRO 194, GLN 198, ILE 309, GLY 312, VAL 313, THR 347, TRP 410, LEU418, and PHE 442	[30]	Center	X	106.712
					Y	10.9282
					Z	61.9545
				Dimension	X	44.4237
					Y	23.4932
					Z	25.0000

DHFR	Ciprofloxacin	ALA 9, ILE 16, LYS 54, LYS 55, THR 56, LEU 75, SER 76, ARG 77, GLU 78, ARG 91, SER 92, LEU 93, GLY 117, SER 118, SER 119, and VAL 120	[28]	Center	X	3.2473
					Y	−3.4640
					Z	−18.6398
				Dimension	X	18.0589
					Y	27.1024
					Z	25.0000

#### 2.10.4. ADME/T Analysis

Computational techniques are being used more and more in modern drug design to analyze pharmacokinetics and pharmacokinetic assessments, which include absorption, distribution, metabolism, excretion, and toxicity. New drug development depends heavily on these investigations, which are referred to as ADMET. As the study at pkCSM demonstrates, one popular strategy is looking at the pharmacological properties of substances (http://biosig.unimelb.edu.au/pkcsm/prediction). Additionally, online tools such as SwissADME (http://www.sib.swiss) have become increasingly popular due to their capacity to assess drug likeness, which is frequently addressed using Lipinski′s rule of five (RO5). These parameters, as outlined by Lipinski, can help determine whether a chemical is suitable for oral use. More specifically, a chemical must meet specific requirements to be deemed orally viable, including having a molecular weight of less than 500 amu, less than 10 hydrogen bond acceptor sites, and a lipophilicity value (Log*p*) of ≤ 5 [33].

#### 2.10.5. Statistical Analysis

The mean ± SEM (standard error of the mean) was utilized to denote the results, which were analyzed using Microsoft Excel and IBM SPSS 27. Dunnett′s test and ANOVA were utilized to evaluate the statistical significance between the control and treatment groups. The result was considered statistically significant if the *p* value remained less than 0.05.

## 3. Results

### 3.1. Isolated Phytochemicals From *J. gendarussa*

The crude extract of *J. gendarussa* leaves was refined using repetitive chromatographic separation and screening to isolate and characterize three components (Figure 1). The structural information of these compounds was elucidated as lupeol (1) [34], *β*‐sitosterol (2) [35], and 1‐monostearin (3) [36] by comparing the proton NMR spectral data to the published values.

Lupeol (1): Compound 1 was isolated as an amorphous solid. The ^1^H NMR spectrum of Compound 1 displayed a doublet of doublets at *δ* 3.20 ppm (*J* = 3.2, 3.6 Hz), corresponding to the proton at Position H‐3. The spectrum also exhibited a deshielded signal at *δ* 2.38 ppm (dt, *J* = 4.8, 4.8 Hz), attributed to H‐19, along with a multiplet at *δ* 1.95 ppm assigned to H‐21. Several singlets appeared in the upfield region, including those at *δ* 0.962 (H‐23), 0.782 (H‐24), 0.852 (H‐25), 1.028 (H‐26), 0.993 (H‐27), and 0.824 (H‐28), corresponding to methyl protons. Notably, two singlets at *δ* 4.708 and 4.588 ppm represented the olefinic protons of H‐29, while another singlet at *δ* 1.591 ppm was allocated to H‐30. The presence of multiple singlet methyl groups, along with the olefinic signals, suggested a sterically hindered structure, potentially indicative of a triterpenoid or steroidal framework. According to the above spectrum data, Compound 1 was identified as lupeol.

*β*‐Sitosterol (2): The ^1^H NMR spectrum of Compound 2, separated as a white amorphous solid, revealed a multiplet at *δ* 3.54 ppm corresponding to the H‐3 proton, indicative of a hydroxylated or oxygenated methine environment. A characteristic downfield doublet at *δ* 5.35 ppm (*J* = 5.2 Hz) indicated the presence of an olefinic proton at H‐6. The upfield region showed multiple methyl group resonances, including a singlet at *δ* 0.693 ppm for H‐18, a singlet at *δ* 1.024 ppm for H‐19, and a doublet at *δ* 0.92 ppm (*J* = 6.0 Hz) corresponding to H‐21. Additional signals included a triplet at *δ* 0.838 ppm (*J* = 8.5 Hz, H‐26), a doublet at *δ* 0.848 ppm (*J* = 5.0 Hz, H‐27), and another triplet at *δ* 0.858 ppm (*J* = 5.0 Hz, H‐29). The presence of an olefinic proton and multiple methyl singlets suggested a steroidal core, possibly a lanostane or stigmastane derivative. According to this spectrum data, Compound 2 was recognized as *β*‐sitosterol.

1‐Monostearin (3): Compound 3 was isolated as a yellowish liquid. The ^1^HNMR spectrum of Compound 3 displayed a triplet at *δ* 0.895 ppm (*J* = 7.6 Hz), corresponding to the terminal methyl group (H‐1), indicative of an aliphatic chain. The existence of a deshielded doublet at *δ* 1.632 ppm (*J* = 5.6 Hz) and a triplet at *δ* 2.346 ppm (*J* = 7.2 Hz) assigned to H‐16 and H‐17, respectively, suggested the presence of a methylene unit adjacent to an electron‐withdrawing functional group. A set of deshielded resonances at *δ* 4.20 ppm (d, *J* = 3.6 Hz, H‐19), 3.98 ppm (m, H‐20), and 3.66 ppm (m, H‐21) likely corresponded to oxygenated methylene and methine protons. These features suggested that Compound 3 contained an ether or ester functional group within its aliphatic framework, possibly indicative of a fatty acid derivative or an esterified alcohol. According to the above spectrum data, Compound 3 was identified as 1‐monostearin.

### 3.2. Assessment of Antioxidant Activity

#### 3.2.1. Measurement of DPPH Scavenging Activity

By comparing the results to the DPPH free radical, the antioxidant potential of various fractionates of the extract of *J. gendarussa* was assessed, with BHT serving as the standard (23.159 *μ*g/mL). The JGESF exerted the most influential free radical scavenging activity (IC_50_ = 24.207 * μ*g/mL), followed by JGDSF with an IC_50_ of 32.093 *μ*g/mL. Conversely, the JGHSF exhibited the lowest antioxidant activity, yielding an IC_50_ value of 58.862 *μ*g/mL (Table 2).

**Table 2:** IC_50_ values of the standard and partitions of leaves of *J. gendarussa*.

**Test material**	**DPPH free radical scavenging activity (IC**_**50**_ ***μ*g/mL)**
BHT	23.159
JGHSF	58.862
JGDSF	32.093
JGESF	24.207

Abbreviations: BHT, tert‐butyl‐1‐hydroxytoluene; JGDSF, dichloromethane soluble fraction of *J. gendarussa* leaves; JGESF, ethyl acetate soluble fraction of *J. gendarussa* leaves; JGHSF, n‐hexane soluble fraction of *J. gendarussa* leaves.

#### 3.2.2. Total Phenolic Content

The total phenolic content of JGDSF and JGESF was measured at 31.394 and 31.242 mg of GAE/gram of extract, respectively. Conversely, the JGHSF demonstrated the lowest phenolic concentration of 12.548 mg of GAE/gram of extract (Table 3).

**Table 3:** The quantitative analysis of the total phenolic content of different fractions of *J. gendarussa* leaves.

**Test material**	**Total phenolic content (mg of GAE/g of dried extract)**	**Regression line**
JGHSF	12.548	*y* = 0.0429*x* − 0.0183 *R* ^2^ = 0.9569
JGDSF	31.394	
JGESF	31.242	

Abbreviations: JGDSF, dichloromethane soluble fraction of *J. gendarussa* leaves; JGESF, ethyl acetate soluble fraction of *J. gendarussa* leaves; JGHSF, n‐hexane soluble fraction of *J. gendarussa* leaves.

### 3.3. Assessment of Analgesic Activity

#### 3.3.1. Central Analgesic Activity

The methanolic crude extract of *J. gendarussa* unraveled substantial central analgesic activity at 200, 400, and 600 mg/kg b.w. doses. Although the obtained result was highly significant almost all the time, the highest 233.47% of time elongation compared to the control was observed after 90 min at a 600 mg/kg b.w. dose (Table 4).

**Table 4:** The central analgesic activity of methanolic crude extract of *J. gendarussa*.

**Group code**	**After 30 min**		**After 60 min**		**After 90 min**	
	**Avg. time of tail immersion**	**% of time elongation**	**Avg. time of tail immersion**	**% of time elongation**	**Avg. time of tail immersion**	**% of time elongation**
CTL	2.25 ± 0.13		2.32 ± 0.17		2.52 ± 0.04	
STD	5.87 ± 0.03^∗∗∗^	161.18	9.83 ± 0.02^∗∗∗^	323.49	17.25 ± 0.1^∗∗∗^	585.20
JGME 1	3.27 ± 0.13^∗∗^	45.49	4.39 ± 0.09^∗∗∗^	89.44	5.695 ± 0.11^∗∗∗^	126.22
JGME 2	4.22 ± 0.09^∗∗∗^	87.65	5.26 ± 0.14^∗∗∗^	126.62	6.81 ± 0.22^∗∗∗^	170.51
JGME 3	5.09 ± 0.07^∗∗∗^	126.59	6.38 ± 0.14^∗∗∗^	175.00	8.395 ± 0.09^∗∗∗^	233.47

*Note:* Average time of tail immersion represented as mean ± SEM (*n* = 4).

Abbreviations: CTL, negative control group receiving 1% Tween 80 in normal saline (10 mL/kg b.w. dose); JGME 1, methanolic crude extract at 200 mg/kg b.w. dose; JGME 2, methanolic crude extract at 400 mg/kg b.w. dose; JGME 3, methanolic crude extract at 600 mg/kg b.w. dose; STD, positive control group receiving diclofenac sodium as standard drug (5 mg/kg b.w. dose).

^∗^ *p* < 0.05,  ^∗∗^ *p* < 0.01, and  ^∗∗∗^ *p* < 0.001 significance level in comparison to the control.

#### 3.3.2. Peripheral Analgesic Activity

The statistical analysis brought out the considerable peripheral analgesic action at dosages of 200, 400, and 600 mg/kg. The maximum peripheral analgesic activity was found at a dose of 600 mg/kg b.w., with an inhibition of 61.96%. In contrast, the conventional DS (5 mg/kg b.w.) inhibited 79.35% (Table 5).

**Table 5:** The peripheral analgesic activity of methanolic crude extract of *J. gendarussa*.

**Group code**	**Mean writhing** **M** **e** **a** **n** ± **S** **E** **M**	**% inhibition of writhing**
CTL	23.0 ± 0.935^∗∗∗^	
STD	4.75 ± 0.415^∗∗∗^	79.35
JGME 1	13 ± 1.023^∗∗∗^	44.57
JGME 2	10 ± 0.612^∗∗∗^	57
JGME 3	8.75 ± 0.739^∗∗∗^	61.96

*Note:* Average writhing represented as mean ± SEM (*n* = 4).

Abbreviation: CTL, negative control group receiving 1% Tween 80 in normal saline; JGME 1, methanolic crude extract at 200 mg/kg b.w. dose; JGME 2, methanolic crude extract at 400 mg/kg b.w. dose; JGME 3, methanolic crude extract at 600 mg/kg b.w. dose; STD, positive control group receiving diclofenac sodium as standard drug (5 mg/kg b.w. dose).

^∗^ *p* < 0.05,  ^∗∗^ *p* < 0.01, and  ^∗∗∗^ *p* < 0.001 significance level in comparison to the control.

### 3.4. Assessment of Antidiarrheal Activity

After 2 h of castor oil administration to mice, the crude methanolic extract at dosages of 200, 400, and 600 mg/kg b.w. demonstrated significant antidiarrheal activity. However, the effect waned over time. After 3 h, antidiarrheal properties were demonstrated at 400 and 600 mg/kg b.w. doses, but no substantial activity was obtained after 4 h of introduction (Table 6).

**Table 6:** The antidiarrheal activity of methanolic extract of *J. gendarussa*.

**Group code**	**After 2 h**		**After 3 h**		**After 4 h**	
	**Total no. of diarrheal feces (** **m** **e** **a** **n** ± **S** **E** **M** **)**	**% reduction of diarrhea**	**Total no. of diarrheal feces (** **m** **e** **a** **n** ± **S** **E** **M** **)**	**% reduction of diarrhea**	**Total no. of diarrheal feces (** **m** **e** **a** **n** ± **S** **E** **M** **)**	**% reduction of diarrhea**
CTL	3.25 ± 0.25		4.25 ± 0.48		2.75 ± 0.25	
STD	0.5 ± 0.29^∗∗∗^	84.62	1.25 ± 0.25^∗∗^	70.59	1.25 ± 0.25^∗∗^	54.55
JGME 1	1.5 ± 0.29^∗∗^	53.85	4.25 ± 0.63	0	2.25 ± 0.48	18.18
JGME 2	1.25 ± 0.48^∗^	61.54	2.75 ± 0.25^∗^	35.29	2.25 ± 0.25	18.18
JGME 3	1 ± 0.41^∗∗^	69.23	1.25 ± 0.63^∗∗^	70.59	1.75 ± 0.48	36.36

*Note:* Total number of diarrheal feces represented as mean ± SEM (*n* = 4).

Abbreviations: CTL, negative control group receiving 1% Tween 80 in normal saline; JGME 1, methanolic crude extract at 200 mg/kg b.w. dose; JGME 2, methanolic crude extract at 400 mg/kg b.w. dose; JGME 3, methanolic crude extract at 600 mg/kg b.w. dose; STD, positive control group receiving loperamide as standard drug at 50 mg/kg b.w. dose.

^∗^ *p* < 0.05,  ^∗∗^ *p* < 0.01, and  ^∗∗∗^ *p* < 0.001 significance in comparison to the control.

### 3.5. Evaluation of Hypoglycemic Activity

The results revealed significant hypoglycemic activity across all doses, except for the 600 mg/kg dose during the first 2 h. Notably, the hypoglycemic effect was noticed to get more pronounced over time rather than dose‐dependent. The highest hypoglycemic activity, with a 48.837% decrease in blood glucose level 3 h after the glucose administration, was noticed with a dose of 400 mg/kg of b.w., whereas the standard glibenclamide exhibited maximum activity 3 h after glucose administration with a reduction rate of 71.059% (Table 7).

**Table 7:** The hypoglycemic activity of methanolic extract of *J. gendarussa*.

**Group code**	**After 1 h**		**After 2 h**		**After 3 h**	
	**BGL**	**% reduction in BGL**	**BGL**	**% reduction in BGL**	**BGL**	**% reduction in BGL**
CTL	14.85 ± 1.49		11.33 ± 0.99		9.68 ± 0.8	
STD	10.73 ± 0.98	27.778	5.85 ± 0.49^∗∗^	48.322	2.8 ± 0.19^∗∗∗^	71.059
JGME 1	10.08 ± 0.64^∗^	32.155	7.25 ± 1.05^∗^	35.982	5.48 ± 0.59^∗∗^	43.410
JGME 2	10.38 ± 0.36^∗^	30.135	7.9 ± 0.81^∗^	30.243	4.95 ± 0.85^∗∗^	48.837
JGME 3	17.88 ± 2.59	−20.370	9.88 ± 1.14	12.804	6.38 ± 1.04^∗^	34.109

*Note:* BGL represented as mean ± SEM (*n* = 4).

Abbreviations: BGL, blood glucose level; CTL, negative control group receiving 1% Tween 80 in normal saline; JGME 1, methanolic crude extract at 200 mg/kg b.w. dose; JGME 2, methanolic crude extract at 400 mg/kg b.w. dose; JGME 3, methanolic crude extract at 600 mg/kg b.w. dose; STD, positive control group receiving glibenclamide as standard drug at 10 mg/kg b.w. dose.

^∗^ *p* < 0.05,  ^∗∗^ *p* < 0.01, and  ^∗∗∗^ *p* < 0.001 significance level in comparison to the control.

### 3.6. Evaluation of Antimicrobial Activity

Various fractions of the crude methanolic extract of *J. gendarussa* exhibit antimicrobial activities against multiple species of bacteria and fungi. The hexane‐soluble fractions of the crude methanolic extract demonstrated antimicrobial activity on *Staphylococcus aureus*, *Shigella dysenteriae*, and *Aspergillus niger*. The dichloromethane‐soluble fractions were active against *Bacillus cereus*, *Staphylococcus aureus*, *Sarcina lutea*, and *Salmonella paratyphi* whereas ethyl acetate showed antimicrobial property on *Sarcina lutea* (Table 8).

**Table 8:** The antimicrobial properties of various fractions of the crude methanolic extract of *J. gendarussa*.

**Types**	**Test microorganisms**	**Diameter of zone of inhibition (mm)**			
		**Ciprofloxacin**	**JGHSF**	**JGDSF**	**JGESF**
Gram‐positive bacteria	*Bacillus cereus*	30	0	15	0
	*Bacillus megaterium*	25	0	0	0
	*Bacillus subtilis*	25	0	0	0
	*Staphylococcus aureus*	35	15	12	0
	*Sarcina lutea*	30	0	9	11

Gram‐negative bacteria	*Salmonella typhi*	31	0	0	0
	*Pseudomonas aeruginosa*	35	0	0	0
	*Salmonella paratyphi*	40	0	11	0
	*Escherichia coli*	32	0	0	0
	*Vibrio mimicus*	33	0	0	0
	*Shigella dysenteriae*	40	20	0	0
	*Pseudomonas anguilliseptica*	32	0	0	0
	*Shigella boydii*	28	0	0	0

Fungi	*Saccharomyces cerevisiae*	32	0	0	0
	*Candida albicans*	30	0	0	0
	*Aspergillus niger*	30	12	0	0

### 3.7. Molecular Docking Analysis for Pharmacological Actions

To determine the potential pharmacological effects of extracts and various solvent fractions obtained from *J. gendarussa*, molecular docking against respective molecular receptors was performed with the help of suitable computer applications. The docking scores gathered from PyRx are all totaled and presented in Table 9, giving a thorough breakdown of the nature, type, and bond distance of the amino acids interacting with ligand atoms. Stronger bindings are the result of lower binding affinities. Extrapolating binding affinities with a null RMSD (root mean square deviation) revealed the optimal docking prediction. The inhibitory property of these isolated compounds on enzymes/receptors was delineated as follows.

**Table 9:** The in silico docking scores derived from the molecular interactions of the separated components from the crude extract of *J. gendarussa* regarding binding affinity (kcal/mol) and the standard drugs BHT, morphine, diclofenac, loperamide, glibenclamide, and ciprofloxacin during the interaction with glutathione reductase (PDB ID: 3GRS), kappa opioid receptor (PDB ID: 6VI4), glucose transporter 3 [GLUT 3] (PDB ID: 4ZWB), and dihydrofolate reductase [DHFR] (PDB ID: 4M6J), mu‐opioid receptor (PDB ID: 5C1M), cyclooxygenase‐2 [COX‐2] (PDB ID: 1CX2) for evaluating the antioxidant, analgesic, antidiarrheal, hypoglycemic, and antimicrobial activities, respectively.

**Com. no.**	**Name of compounds/drugs**	**PubChem CID**	**Receptors/macromolecules′ binding affinity (kcal/mol)**					
			**3GRS**	**5C1M**	**1CX2**	**6VI4**	**4ZWB**	**4M6J**
C1	Lupeol	259846	−8.2	−6.5	−2.9	−7.3	−8.6	−6.6

C2	*β*‐Sitosterol	222284	−8.6	−10	−4.3	−7.7	−9.8	−7.9

C3	1‐Monostearin	24699	−5.8	−6.1	−7.2	−4.6	−6.5	−5.4

Standard drugs	BHT	31404	−5.8					
	Morphine	5288826		−7.7				
	Diclofenac	3033			−7.7			
	Loperamide	3955				−7.3		
	Glibenclamide	3488					−9.3	
	Ciprofloxacin	2764						−8.2

#### 3.7.1. Antioxidant Activity by 3GRS Inhibition

Maintaining the balance between oxidative stress and redox homeostasis depends on the interaction with the 3GRS enzyme. The 3GRS enzyme and each compound (1–3) demonstrated prominent interactions, with higher binding affinities (−5.8 to −8.6 kcal/mol) than the standard ligand BHT (−5.8 kcal/mol) (Table 9). *β*‐Sitosterol (Compound C2) exhibited the highest binding affinity to the 3GRS protein, followed by lupeol (Compound C1), 1‐monostearin (Compound C3), and BHT. Figures 2a, 3a, and 4a present the active binding sites of the 3GRS enzyme during its molecular engagement with the separated substances. During compound C2 (*β*‐sitosterol) interactions with the protein, one conventional hydrogen bond and four hydrophobic bonds (four alkyl) were revealed, demonstrating the most binding interactions (−8.6 kcal/mol). Lupeol seemed to have a total of 10 binding interactions with the 3GRS protein. There were five conventional hydrogen bonds and five hydrophobic bonds among them; two were alkyl and three were Pi–alkyl interactions. Again, among the four hydrogen bonds that are seen between 1‐monostearin and the target protein, 3GRS, two were conventional hydrogen bonds and the rest two were carbon–hydrogen bonds. Five hydrophobic bonds (three alkyl and two pi‐alkyl) were also revealed during the binding interactions of 1‐monostearin and 3GRS (Table S1).

Figure 2Graphical presentation of the best binding interactions (2D and 3D) of (a) (3GRS + lupeol), (b) (5C1M + lupeol), (c) (1CX2 + lupeol), (d) (6VI4 + lupeol), (e) (4ZWB + lupeol), and (f) (4M6J + *β*‐lupeol).

**Figure:**
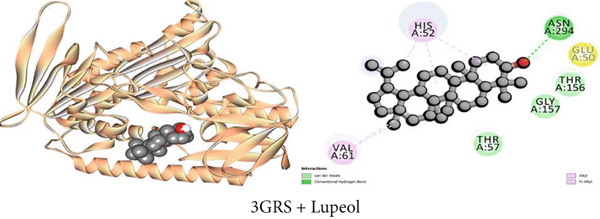
(a)

**Figure:**
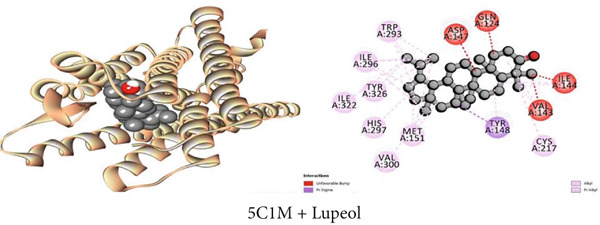
(b)

**Figure:**
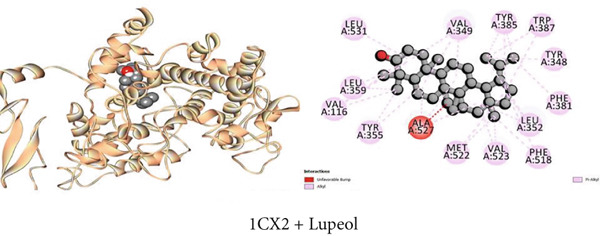
(c)

**Figure:**
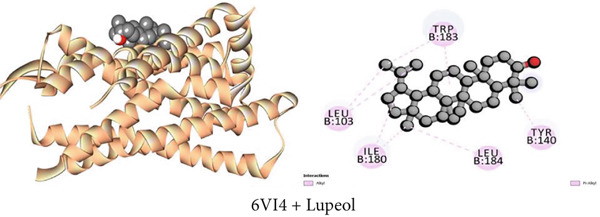
(d)

**Figure:**
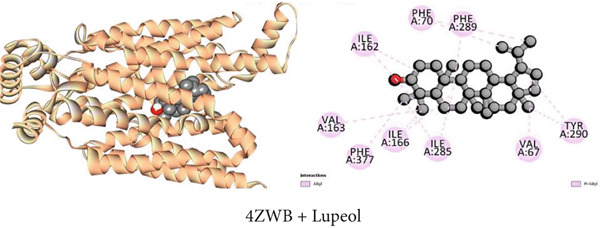
(e)

**Figure:**
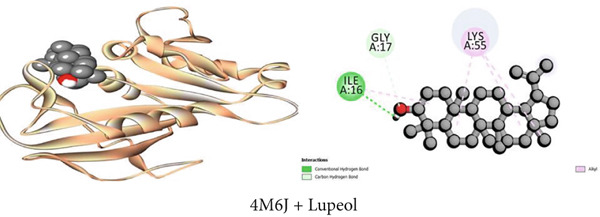
(f)

Figure 3Graphical presentation of the best binding interactions (2D and 3D) of (a) (3GRS + *β*‐sitosterol), (b) (5C1M + *β*‐sitosterol), (c) (1CX2 + *β*‐sitosterol), (d) (6VI4 + *β*‐sitosterol), (e) (4ZWB + *β*‐sitosterol), and (f) (4M6J + *β*‐sitosterol).

**Figure:**
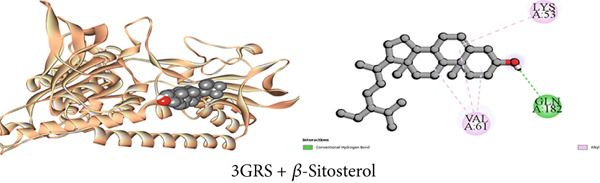
(a)

**Figure:**
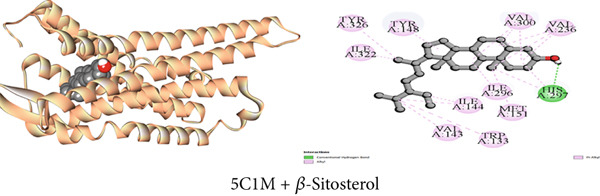
(b)

**Figure:**
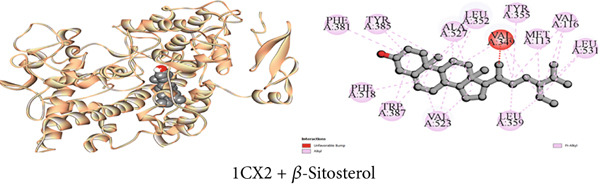
(c)

**Figure:**
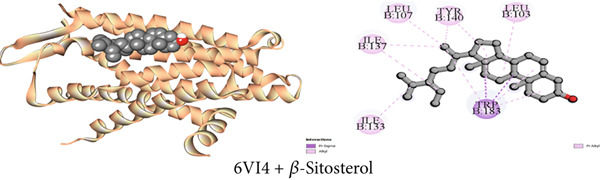
(d)

**Figure:**
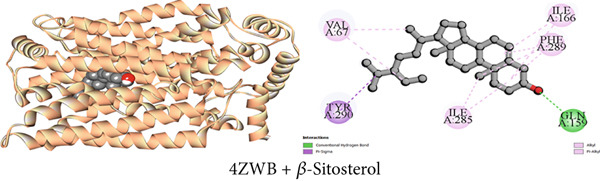
(e)

**Figure:**
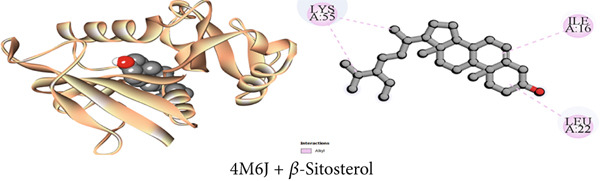
(f)

Figure 4Graphical presentation of the best binding interactions (2D and 3D) of (a) (3GRS + 1‐monostearin), (b) (5C1M + 1‐monostearin), (c) (1CX2 + 1‐monostearin), (d) (6VI4 + 1‐monostearin), (e) (4ZWB + 1‐monostearin), and (f) (4M6J + 1‐monostearin).

**Figure:**
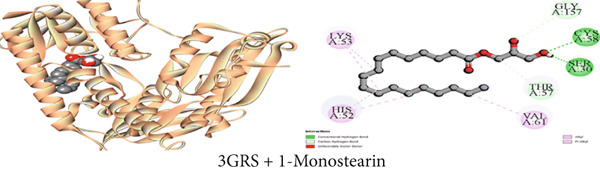
(a)

**Figure:**
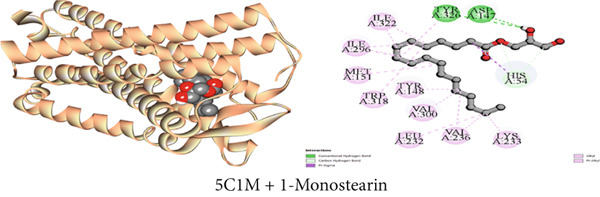
(b)

**Figure:**
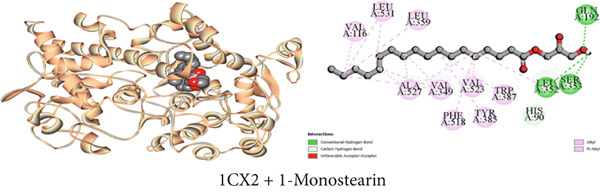
(c)

**Figure:**
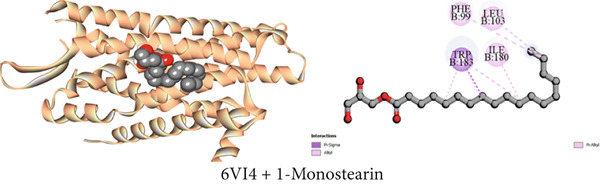
(d)

**Figure:**
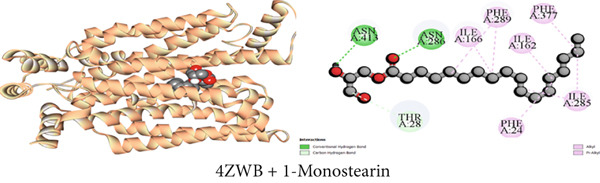
(e)

**Figure:**
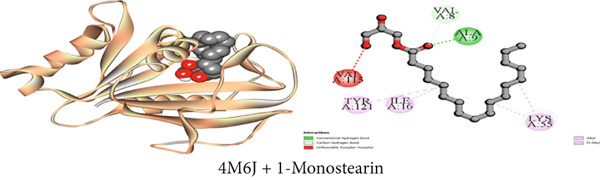
(f)

#### 3.7.2. Central Analgesic Activity by Mu‐Opioid Receptor Inhibition

Molecular docking with the mu‐opioid receptor was carried out to elucidate the molecular interaction of the central analgesic effect of the isolated compounds. Compared to the traditional central analgesic medication morphine, Compound C2 (*β*‐sitosterol) exhibits a higher binding affinity (−10 kcal/mol) for the mu‐opioid receptor (−7.7 kcal/mol). The docking scores of the isolated compounds against the protein were determined in the sequence *β*‐sitosterol > morphine > lupeol > 1‐monostearin (Table 9). Figures 2b, 3b, and 4b illustrate the interaction with distinct phytoconstituents with the mu‐opioid receptor at its active binding sites. A total of 18 binding interactions were noticed between *β*‐sitosterol and the mu‐opioid receptor. Among the interactions, one was a conventional hydrogen bond and the rest were 17 hydrophobic interactions (12 alkyl and five pi‐alkyl interactions). Lupeol seemed to have 24 hydrophobic interactions (one pi‐sigma, 16 alkyl, and seven pi‐alkyl interactions) whereas 1‐monostearin had four hydrogen bonds (two conventional hydrogen bonds and two carbon‐hydrogen bonds) and 13 hydrophobic interactions (one pi‐sigma, nine alkyl, and three pi‐alkyl interactions) with the mu‐opioid receptor (Table S2).

#### 3.7.3. Peripheral Analgesic Activity by COX‐2 Protein Inhibition

Table 9 reveals that the interaction between *J. gendarussa* crude extracts and the COX‐2 protein was the basis for the molecular mechanism predicting the analgesic impact on the peripheral nerve. A comparative evaluation with the typical analgesic medication diclofenac indicated that every chemical exhibited an affinity for binding the COX‐2 enzyme. The binding affinities toward COX‐2 protein are as follows: DS > 1‐monostearin > *β*‐sitosterol > lupeol (Table 9). The hydrophobic properties of the compounds characterize the most binding interaction locations. Compound C3 (1‐monostearin) displayed 15 hydrophobic contacts (12 alkyl and three pi‐alkyl interactions), two carbon‐hydrogen bonds, and three conventional hydrogen bonds. Again, *β*‐sitosterol possessed 26 hydrophobic interactions (19 alkyl and seven pi‐alkyl) whereas lupeol exhibited 30 hydrophobic contacts (19 alkyl and 11 pi‐alkyl) with the COX‐2 protein (Table S3). Figures 2c, 3c, and 4c provide an extensive list of all interacting amino acids and their three‐letter codes.

#### 3.7.4. Antidiarrheal Activity by Kappa Opioid Receptor Inhibition

Using molecular docking, the study focused on how the kappa opioid receptor interacts with its molecules. The most potent antidiarrheal effect was exhibited by Compound C2 (*β*‐sitosterol), which had a higher affinity (−7.7 kcal/mol) than loperamide (−7.3 kcal/mol). Significant binding affinities were observed for Compounds C3 (1‐monostearin) and C1 (lupeol) at −4.6 and −7.3 kcal/mol, respectively. For example, the receptor‐binding affinities of the compounds may be arranged in the following manner: *β*‐sitosterol > lupeol > loperamide > 1‐monostearin (Table 9). Most binding interaction sites exhibit hydrophobic properties. Table S4 illustrates that Compound C2 exhibited 14 hydrophobic interactions, with two, five, and seven of these interactions being pi‐sigma, alkyl, and pi‐alkyl bonds, respectively. Lupeol had nine hydrophobic contacts while 1‐monostearin had eight of them with the protein (Table S4). Figures 2d, 3d, and 4d display all of the interacting amino acids and their respective three‐letter codes.

#### 3.7.5. Hypoglycemic Activity by GLUT 3 Inhibition

As illustrated in Table 9, the GLUT 3 receptor interactions with three compounds resulted in molecular docking scores of −8.6, −9.8, and −6.5 kcal/mol, respectively. Upon docking with the strongest isolated hypoglycemic Compound C2 (*β*‐sitosterol), 10 hydrophobic interactions, one pi‐sigma, six alkyl, and three pi‐alkyl, as well as one conventional hydrogen bond were noticed as indicated in Table S5. Additionally, lupeol exhibited 14 hydropic contacts (eight alkyl and six pi‐alkyl) and 1‐monostearin exerted two conventional hydrogen bonds, one carbon‐hydrogen bond, and eight hydrophobic interactions (four alkyl and four pi‐alkyl) with GLUT 3 protein (Table S5). Figures 2e, 3e, and 4e contain an extensive list of the protein′s active binding sites when docked with the specified drugs.

#### 3.7.6. Antibacterial Activity by DHFR Inhibition

In comparison to ciprofloxacin as the standard drug (−8.2 kcal/mol), the binding affinities of the detached chemical compounds (1–3) from the plant were −5.4 to −7.9 kcal/mol. The docking scores were arranged in the subsequent order: ciprofloxacin (standard medication) > *β*‐sitosterol > lupeol > 1‐monostearin (Table 9). *β*‐Sitosterol possessed four hydrophobic interactions with the DHFR enzyme while lupeol had one conventional hydrogen bond, one carbon–hydrogen bond, and four alkyl interactions. 1‐Monostearin, on the other hand, possessed two conventional hydrogen bonds, one carbon–hydrogen bond, and four hydrophobic contacts (three alkyl and one pi‐alkyl). The DHFR protein′s bonding profiles with the other detached chemical compounds are extensively represented and summarized in Table S6 and Figures 2f, 3f, and 4f.

#### 3.7.7. Pharmacokinetics, Toxicity, and Drug Likeliness Analysis

Computational studies are currently being employed to model the drug′s pharmacokinetics, encompassing its absorption, distribution, metabolism, excretion, and also toxicity [37]. Computational studies are necessary to see the pharmacokinetics forecast of a chemical because they are a more efficient and economical way. If bioactive molecules with a good ADMET profile are extracted from plants, they can be considered lead compounds [38]. In the absorption parameter, the two factors that must be considered are intestinal absorption and water solubility. Water solubility is a critical factor in medication bioavailability. The optimal range for solubility in water is defined as less than 0 and greater than −0.5, and the compounds′ water solubility falls within the required interval in this study. According to Khan et al.′s research, the optimum average intestinal absorption of a substance exceeds 80% [39]. The distribution of drugs or lead compounds in the body is predicted by three factors: volume distribution, blood–brain barrier (BBB) permeability, and central nervous system (CNS) permeability [40]. Generally, a medicine or molecule has a volume of distribution between 0.5 and 3 L/kg [41]. These three compounds are classified as having inadequate drug distribution in the bloodstream. A medicine is considered effective if it is challenging to infiltrate the CNS or the BBB [42]. Drug absorption into the BBB and CNS is categorized into three classes: high (> 2.0), moderate (0.1–2.0), and low (< 0.1) absorption values [43]. These three isolated compounds are categorized as low absorption since their absorption value is < 0.1. This indicates that the CNS and BBB are challenging for these three chemicals to access. It is possible to predict the metabolism of a medication and ascertain whether it will inhibit CYP enzymes. An enzyme called CYP, or cytochrome 450, is involved in Phase 1 metabolic activities and the digestive system [44]. These three test substances did not exhibit any inhibition of the various categories of CYP enzymes. The excretory system is the final pharmacokinetic characteristic to evaluate, as a more rapid excretion process correlates with an elevated overall clearance value. This has a beneficial impact on the human body [45]. Lupeol′s excretion process is relatively efficient, as evidenced by its high total clearance value compared to other compounds. Toxicology prediction science uses computational techniques to minimize the necessity for toxicity testing on investigational animals, as the outcomes derived from these predictions are comparable to those of in vivo assessments [46, 47]. The toxicity level ratio is determined by the lethal dose value. A crucial parameter that indicates the toxicity of a compound is the lethal dose of 50 (LD_50_) [48]. The LD_50_ of a substance is classified as fatal, toxic, harmful, possibly harmful, or nontoxic based on its concentration in the body. The classification range is as follows: LD_50_ < 50 mg/kg is fatal when ingested, 50 mg/kg ≤ LD_50_ < 300 mg/kg is toxic if ingested, 300 mg/kg ≤ LD_50_ < 2000 mg/kg is harmful when ingested, 2000 mg/kg ≤ LD_50_ < 5000 mg/kg is potentially harmful if swallowed, and LD_50_ > 5000 mg/kg is nontoxic [49, 50]. The Lipinski RO5 is employed in drug‐likeness analysis to assess the chemical and physical properties of a compound for purposes of active drug use [51]. Lipinski′s RO5 satisfies specific criteria, such as a molar refractivity value of 40–130, a Log *p* of less than 5, a number of hydrogen bond acceptors less than 10, a number of hydrogen bond donors less than 10, and a molecular mass less than 500 Dalton [52, 53]. Table 10 indicates that *β*‐sitosterol and lupeol have a single violation of the RO5 rule, whereas 1‐monostearin does not. The rule permits a substance to be administered orally as a medication if it has not been violated more than once [54]. Furthermore, the solubility and permeability of compounds are substantially reduced when they exhibit multiple RO5 violations [55].

**Table 10:** ADME/T prediction of lupeol, *β*‐sitosterol, and 1‐monostearin.

**Properties**	**Model name (unit)**	**Lupeol**	***β*-Sitosterol**	**1-Monostearin**
Absorption	Water solubility	−7.398	−7.565	−5.298
	Intestinal absorption (human)	93.588	92.986	90.825
	Skin permeability	−2.889	−2.802	−2.754

Distribution	VDs (human)	1.092	1.161	0.416
	Fraction unbound (human)	0	0	0.204
	BBB permeability	0.252	0.504	−0.409
	CNS permeability	−0.88	−0.983	−3.253

Metabolism	CYP2D6 substrate	No	No	No
	CYP3A4 substrate	Yes	Yes	No
	CYP1A2 inhibitor	No	No	No
	CYP2C19 inhibitor	No	No	Yes
	CYP2C9 inhibitor	No	No	Yes
	CYP2D6 inhibitor	No	No	No
	CYP3A4 inhibitor	No	No	No

Excretion	Total clearance	0.116	0.591	1.857
	Renal OCT2 substrate	No	No	No

Toxicity	AMES toxicity	No	No	No
	Max. tolerated dose (human)	0.454	0.613	1.283
	hERG I inhibitor	No	No	No
	hERG II inhibitor	Yes	Yes	Yes
	Oral rat acute toxicity (LD50) mol/kg	2.065	2.063	1.611
	Oral rat chronic toxicity (LOAEL)	1.021	0.921	2.779
	Hepatotoxicity	No	No	No
	Skin sensitization	No	No	Yes
	*T. pyriformis* toxicity	0.574	0.858	1.08
	Minnow toxicity	−2.684	−2.939	−2.005

Lipinski rule	Bioavailability score (%)	0.55	0.55	0.55
	Lipinski′s rule of five	Yes; 1 violation: MLOGP > 4.15	Yes; 1 violation: MLOGP > 4.15	Yes; 0 violation

## 4. Discussion

Plant‐derived medicines and natural compounds have cured numerous diseases since the earliest civilizations. Since prehistoric times, all ethnic groups have extensively used plants to enhance health and address various illnesses. According to the World Health Organization (WHO), 80% of the general public depends on traditional medicine as their primary healthcare source [56]. The numerous pharmacological benefits of phenolic phytochemicals, for example, anticarcinogenic, antidiabetic, anti‐inflammatory, cardioprotective, antioxidant, antimicrobial, and antidiarrheal properties, have attracted worldwide attention [57]. Thus, phenolic compounds from plant origin are increasingly recognized as a source of potential therapeutic candidates with novel medications that are more efficacious and less toxic [58]. The present investigation ended with three compounds from the methanolic leaf extract of *J. gendarussa*. These compounds were lupeol (1), *β*‐sitosterol (2), and 1‐monostearin (3).

The onset of a number of diseases, for example, neurological disorders, rheumatoid arthritis, diabetes mellitus, obesity, and cancer, is caused by the overproduction of free radicals in the body [58]. The hydroxyl groups on the phenolic compounds derived from plants enable them to effectively combat free radicals by scavenging ROS [59]. Lupeol (1) is a pentacyclic triterpene that demonstrates antioxidative properties by directly scavenging free radicals and protecting membrane permeability [60]. *β*‐Sitosterol (2) exhibits antioxidant properties without inducing acute and chronic toxicity or overturning any antioxidant enzymes synthesis pathway [61]. The findings indicate a positive association between elevated antioxidant activity and phenolic content across various plant extracts, consistent with prior reports by Jain et al. and Mondal et al. [62, 63]. Among the fractions, the highest phenolic content with the lowest IC_50_ value was seen in the dichloromethane and ethyl acetate soluble fractions, whereas the lowest phenolic content with the highest IC_50_ value was noticed in the HSF. To further support the antioxidant potential, the isolated compounds were docked with 3GRS. According to the molecular docking investigation, *β*‐sitosterol was the prominent one among the molecules to have free radical scavenging property. Lupeol exhibits both enzymatic and nonenzymatic activity by reducing oxidative cellular injury and lipid peroxidation. This type of action may be attributed to hydrophobic interactions between the compound and the 3GRS enzyme [64]. This outcome is also enhanced by *β*‐sitosterol, which is responsible for reducing the detrimental effects of free radicals, such as peroxynitrite, and inhibiting the synthesis of nitric oxide (NO) and lipid peroxidation (LPO) [65, 66]. Consequently, this investigation indicates that *J. gendarussa* may be a potent source of natural antioxidants. Additional investigation is inevitable to have a complete understanding of the primary antioxidant mechanisms of the studied phytochemicals.

Natural analgesics are being explored as substitutes for synthetic drugs due to their reduced side effects [67]. The tail immersion technique was exploited to assess central analgesic activity and the acetic acid–induced writhing test was utilized to investigate the peripheral analgesic activity of *J. gendarussa*. Central analgesics function by increasing pain tolerance and modifying the body′s reaction to pain [68]. The synthesis of prostaglandins and histamine is induced by the intraperitoneal delivery of 1% acetic acid, which in turn triggers the inflammatory response [69]. The analgesia of Swiss albino mice was markedly reduced (*p* < 0.05) by the testing fractions of *J. gendarussa* leaf extract at doses of 200, 400, and 600 mg/kg b.w. in this investigation. They have been functioning through both central and peripheral pathways. Additionally, in the computational analysis that backed up the in vivo results, the substances in the plant extract showed stronger affinity for the mu‐opioid receptor than did the conventional medication morphine. Furthermore, through traditional hydrogen bond and hydrophobic interactions, the *β*‐sitosterol exhibited a greater binding affinity than ordinary morphine [70]. The isolated compound *β*‐sitosterol was also reported to restrict the central opioid receptors or liberate endogenous opioid peptides and limit the generation of bradykinins and prostaglandins. These findings affirm the analgesic properties of the plant extract which could be a source of natural analgesics upon further investigation to unveil its therapeutic efficacy.

In developing nations, diarrhea is a major cause of mortality, primarily affecting infants and young children. Traditionally, medicinal herbs have been utilized for dealing with various gastrointestinal illnesses, including diarrhea. Disequilibrium between absorption patterns and gastrointestinal motility of the intestinal tract can result in diarrhea [71]. Castor oil carries ricinoleic acid, which is reported to induce peristaltic movement, which may result in diarrhea, by producing prostaglandins that irritate the intestinal membrane [72]. Phytoconstituents, including flavonoids and tannins, have also been reported to have antidiarrheal properties [73]. The antidiarrheal effects observed in animal models may also be attributed to specific alkaloids, mono‐, di‐, and tri‐terpenoids, and cardiac glycosides found in plants [74, 75]. Consequently, this investigation assessed the safety and efficacy of *J. gendarussa* as an antidiarrheal treatment for diarrheal disease. Statistically significant antidiarrheal activity was observed in the second hour at doses of 200 mg/kg b.w. and in the second and third hours at doses of 600 mg/kg b.w. of castor oil administration in the current study. Nevertheless, the methanolic crude extract at a dose of 600 mg/kg b.w. of *J. gendarussa* displayed the highest antidiarrheal efficacy. The computational simulation study revealed significant binding scores for isolated *β*‐sitosterol, 1‐monostearin, and lupeol in contrast to loperamide as the standard drug. Results from visualization and docking analysis indicate that the separated chemicals have a network of chemical interactions with the targeted enzymes. *β*‐Sitosterol, one of the isolated molecules, exhibited 14 hydrophobic interactions with the kappa opioid receptor and had the highest docking score (Table 9 and Table S4). Lupeol also binds to the opioid receptor with a high affinity which necessitates further investigations on this plant to reveal its antidiarrheal potential.

Furthermore, the potential to reduce blood sugar levels exists in phenolic compounds extracted from medicinal plants, either independently or in conjunction with steroids, alkaloids, terpenes, and other plant constituents. Hence, these can help develop alternative strategies for managing diabetes as they are less costly and possess lower side effects [76, 77]. For the first 3 h following glucose loading, the methanolic crude extract of the *J. gendarussa* leaf exhibited statistically significant hypoglycemic effects at concentrations of 200‐, 400‐, and 600‐mg/kg b.w. Nevertheless, the methanolic crude extract revealed the most potent hypoglycemic activity at a dose of 400 mg/kg b.w. after 3 h of administering glucose. The in vivo findings on hypoglycemic activity of the plant extracts at various doses have been verified by the in silico study. *β*‐Sitosterol was found to have the most substantial hypoglycemic impact (−9.8 kcal/mol) among all the extracted substances in the molecular modeling investigation, and it also had a greater binding affinity in the direction of the GLUT 3 protein (Table 9). This investigation tried to find out how *β*‐sitosterol affected the production of insulin signaling molecules in the fat tissue of rats that were fed a high‐fat diet and sucrose to cause Type 2 diabetes [78]. Additional isolated polyphenols also showed significant binding affinities with the GLUT 3 protein, suggesting hypoglycemic potential. Consequently, *J. gendarussa* may be considered a possible source of hypoglycemic phytoconstituents based on the findings of the current investigation.

Bacteria possess a genetic capacity for gaining resistance [79]. Consequently, the pharmaceutical industry has developed a variety of antibiotics, but there is a necessity to identify new antimicrobial compounds that can effectively combat a broad spectrum of microorganisms [80]. The specified microorganisms were subjected to an antimicrobial potential assessment of the extracts from *J. gendarussa* leaves. The presence of zones of inhibition (Table 8) suggested that specific extracts were efficacious against both bacteria and fungi. Ciprofloxacin′s zones of inhibition were 25–40 mm, while the plant extracts′ zones of inhibition against bacteria varied from 9 to 20 mm (Table 8). The zone of inhibition for fungi was observed to be 9–15 mm for plant extracts, whereas it was seen to be 16–18 mm by the antifungal agent fluconazole. Several studies also confirm the significant antimicrobial activity of *J. gendarussa* leaf extracts [81–84], whereas the antibacterial and antifungal properties were found with zone of inhibition values ranging from 10 to 13 mm for both [82]. These findings are also in line with the results of other promising plant extracts with potent antimicrobial properties. For example, *Nigella sativa*, a promising antimicrobial agent, showed antibacterial and antifungal activities with inhibitory zones measuring from 11.5 to 15.1 mm and 13–16.3 mm, respectively [85]. In computational analysis, DHFR and *β*‐sitosterol combine with each other, creating four alkyl bonds with a strong binding value of −7.9 kcal/mol. This is higher than the reference binding value of 8.2 kcal/mol that ciprofloxacin has. Conversely, there are two regular hydrogen bonds, one carbon–hydrogen link, three alkyl bonds, and an unfavorable acceptor–acceptor bond that hold 1‐monosterein together with a binding score of −5.4 kcal/mol (Table 9 and Table S6). Therefore, this plant extract can be considered a potential antimicrobial agent with further studies to confirm its efficacy and safety.

Nevertheless, the ADME/T study has certain drawbacks. The quality of pkCSM′s and SwissADME′s experimental data is a limitation that could lead to errors when working with insufficient datasets. These technologies, which are dependent on oversimplified models, are incapable of predicting unanticipated chemical responses, nor are they capable of accounting for ongoing changes in biological systems or species‐specific variations. The physicochemical parameter specifications are broad guidelines, and differences may arise between different computational instruments. That means an extended ADME/T investigation is advised to determine such compounds′ drug‐like eligibility, particularly in a clinical environment.

In spite of the insightful findings using both experimental and computational approaches, this research investigation certainly has some limitations. A limitation of this study is that the weight loss of the mice was not taken into account during the antidiarrheal assay. We acknowledge this oversight and will ensure the inclusion of this parameter in future experiments for a more comprehensive assessment. Again, the investigation lacks addressing certain research questions which need to be addressed in future investigations. A comprehensive phytochemical profiling needs to be established using more advanced research facilities like UPLC‐MS/MS or carbon NMR as well as 2D NMR. A toxicological study should be done to establish the safety profile of the isolated molecules that was not done in the study and can be considered a limitation of this study. A robust SAR model might be established to determine the important functional groups or molecular structures within the structure and pave the way to modify and enhance their pharmacological properties. Again, the in vivo experiments can also be carried out with larger sample sizes and a varied number of doses to explore its potential more precisely.

## 5. Conclusion

Three promising bioactive compounds were purified and characterized from the methanolic crude extracts of *J. gendarussa* Burm. f. leaves which demonstrate prominent binding affinities with therapeutic targets in a molecular docking study. Additionally, this methanolic extract can be an opportunistic source of potential bioactive compounds possessing antioxidant, analgesic, hypoglycemic, and antidiarrheal properties. However, it is imperative to conduct further investigation to isolate additional bioactive constituents of *J. gendarussa* and to verify the plant′s reported pharmacological properties in accordance with the current evidence.

## Ethics Statement

All the experiments were accomplished following the Declaration of Helsinki and the Animal Scientific Procedures Act, 1986. The protocol and all ethical aspects were thoroughly reviewed and approved by the Animal Ethics Committee of the State University of Bangladesh (Ref. 2025‐01‐05/SUB/I‐ERC/001).

## Consent

The authors have nothing to report.

## Conflicts of Interest

The authors declare no conflicts of interest.

## Author Contributions

Farzana Akter Munny, Mehedi Islam, Md. Rabiul Islam, and Md. Aslam Hossain: conceptualization, data curation, writing—original draft, writing—review and editing. Mahafuza Akter, Mushtahsin Ferdousi, Md. Moaz Ahmed Asif, and Md. Solaiman Hossain: formal analysis, methodology. Sabrina Sharmin, Md Zahidul Islam: project administration, investigation. Farzana Akter Munny and Mehedi Islam contributed equally to this work.

## Funding

No funding was received for this manuscript.

## Supporting information


**Supporting Information** Additional supporting information can be found online in the Supporting Information section.

## Data Availability

The data that support the findings of this study are available from the corresponding authors upon reasonable request.

## References

[1] Gozubuyuk G. S. , Aktas E. , and Yigit N. , An Ancient Plant *Lawsonia inermis* (Henna): Determination of In Vitro Antifungal Activity Against Dermatophytes Species, Journal de Mycologie Médicale. (2014) 24, no. 4, 313–318, 10.1016/j.mycmed.2014.07.002, 2-s2.0-84916633156, 25442917. 25442917

[2] Harvey A. L. , Natural Products in Drug Discovery, Drug Discovery Today. (2008) 13, no. 19-20, 894–901, 10.1016/j.drudis.2008.07.004, 2-s2.0-52049109838, 18691670. 18691670

[3] Thomas T. D. and Yoichiro H. , In Vitro Propagation for the Conservation of a Rare Medicinal Plant *Justicia gendarussa* Burm. f. by Nodal Explants and Shoot Regeneration From Callus, Acta Physiologiae Plantarum. (2010) 32, no. 5, 943–950, 10.1007/s11738-010-0482-1, 2-s2.0-78049266245.

[4] Yadav A. K. , Saraswat S. , Sirohi P. , Rani M. , Srivastava S. , Singh M. P. , and Singh N. K. , Antimicrobial Action of Methanolic Seed Extracts of *Syzygium cumini* Linn. on *Bacillus subtilis* , AMB Express. (2017) 7, no. 1, 10.1186/s13568-017-0500-4, 2-s2.0-85032908905, 29098477. PMC5668226 29098477

[5] Shamili G. and Santhi G. , Identification and Characterization of Bioactive Compounds of Leaves of Justicia gendarussa Burm. F., International Journal of Scientific Research in Biological Sciences. (2019) 6, no. 1, 145–153, 10.26438/ijsrbs/v6i1.145153.

[6] Paval J. , Kaitheri S. K. , Potu B. K. , Govindan S. , Kumar R. S. , Narayanan S. N. , and Moorkoth S. , Anti-Arthritic Potential of the Plant *Justicia gendarussa* Burm F., Clinics (São Paulo, Brazil). (2009) 64, no. 4, 357–362, 10.1590/S1807-59322009000400015, 2-s2.0-67650620495, 19488595. 19488595 PMC2694464

[7] Mpiana P. T. , Ngbolua K. N. , Mudogo V. , Tshibangu D. S. T. , Atibu E. K. , Tshilanda D. D. , and Misengabu N. M. , Anti Sickle Erythrocytes Haemolysis Properties and Inhibitory Effect of Anthocyanins Extracts of *Trema orientalis* (Ulmaceae) on the Aggregation of Human Deoxyhemoglobin S *In Vitro* , Journal of Medical Sciences. (2011) 11, no. 3, 129–137, 10.3923/jms.2011.129.137.

[8] Kumar K. S. , Sabu V. , Sindhu G. , Rauf A. A. , and Helen A. , Isolation, Identification and Characterization of Apigenin From *Justicia gendarussa* and Its Anti-Inflammatory Activity, International Immunopharmacology. (2018) 59, 157–167, 10.1016/j.intimp.2018.04.004, 2-s2.0-85045565400, 29655057. 29655057

[9] Ayob Z. , Abd Samad A. , and Mohd Bohari S. P. , Cytotoxicity Activities in Local Justicia gendarussa Crude Extracts Against Human Cancer Cell Lines, Jurnal Teknologi (Sciences & Engineering). (2013) 64, no. 2, 45–52, 10.11113/jt.v64.2043, 2-s2.0-84884737137.

[10] Wardojo B. P. E. , Widiyanti P. , and Herupradoto E. B. A. , The Effect of *Gendarussin* A isolates of *Justicia gendarussa Burm.f*. Leaf in Reverse Transcriptase Inhibition of HIV Type I In Vitro, Indonesian Journal of Tropical and Infectious Disease. (2015) 5, no. 5, 136–141, 10.20473/ijtid.v5i5.307.

[11] Zhang H. J. , Rumschlag-Booms E. , Guan Y. F. , Liu K. L. , Wang D. Y. , Li W. F. , Nguyen V. H. , Cuong N. M. , Soejarto D. D. , Fong H. H. S. , and Rong L. , Anti-HIV Diphyllin Glycosides From *Justicia gendarussa* , Phytochemistry. (2017) 136, 94–100, 10.1016/j.phytochem.2017.01.005, 2-s2.0-85009822203, 28110956. 28110956

[12] Ningsih I. Y. , Purwanti D. I. , Wongso S. , Prajogo B. E. , and Indrayanto G. , Metabolite Profiling of *Justicia gendarussa* Burm. f. Leaves Using UPLC-UHR-QTOF-MS, Scientia Pharmaceutica. (2015) 83, no. 3, 489–500, 10.3797/scipharm.1411-08, 2-s2.0-84942421733, 26839833. 26839833 PMC4727782

[13] Carneiro M. R. B. , Sallum L. O. , Martins J. L. R. , Peixoto J. D. C. , Napolitano H. B. , and Rosseto L. P. , Overview of the *Justicia* Genus: Insights Into Its Chemical Diversity and Biological Potential, Molecules. (2023) 28, no. 3, 10.3390/molecules28031190, 36770856. PMC9920429 36770856

[14] Ayob Z. , Jamil S. , Mohd Bohari S. P. , Ahmad F. , and Abd Samad A. , Detection of Naringenin and Kaempferol in *Justicia gendarussa* Leaf Extracts by GC-FID, Sains Malaysiana. (2017) 46, no. 3, 457–461, 10.17576/jsm-2017-4603-13, 2-s2.0-85019270053.

[15] Panche A. N. , Diwan A. D. , and Chandra S. R. , Flavonoids: An Overview, Journal of Nutritional Science. (2016) 5, e47, 10.1017/jns.2016.41, 2-s2.0-85007481080. 28620474 PMC5465813

[16] Annunziata G. , Maisto M. , Schisano C. , Ciampaglia R. , Daliu P. , Narciso V. , Tenore G. C. , and Novellino E. , Colon Bioaccessibility and Antioxidant Activity of White, Green and Black Tea Polyphenols Extract After In Vitro Simulated Gastrointestinal Digestion, Nutrients. (2018) 10, no. 11, 10.3390/nu10111711, 2-s2.0-85056414604, 30413043. PMC6266738 30413043

[17] Cheng C. S. , Gu Q. H. , Zhang J. K. , Tao J. H. , Zhao T. R. , Cao J. X. , Cheng G. G. , Lai G. F. , and Liu Y. P. , Phenolic Constituents, Antioxidant and Cytoprotective Activities, Enzyme Inhibition Abilities of Five Fractions From *Vaccinium dunalianum* Wight, Molecules. (2022) 27, no. 11, 10.3390/molecules27113432, 35684371. PMC9181978 35684371

[18] Pisoschi A. M. , Pop A. , Cimpeanu C. , and Predoi G. , Antioxidant Capacity Determination in Plants and Plant-Derived Products: A Review, Oxidative Medicine and Cellular Longevity. (2016) 2016, 9130976, 10.1155/2016/9130976, 2-s2.0-85009083801, 28044094. 28044094 PMC5164913

[19] Chakraborty A. J. , Uddin T. M. , Matin Zidan B. M. R. , Mitra S. , das R. , Nainu F. , Dhama K. , Roy A. , Hossain M. J. , Khusro A. , and Emran T. B. , *Allium cepa*: A Treasure of Bioactive Phytochemicals With Prospective Health Benefits, Evidence-Based Complementary and Alternative Medicine. (2022) 2022, 4586318, 10.1155/2022/4586318, 35087593. 35087593 PMC8789449

[20] Elisabetsky E. , Amador T. A. , Albuquerque R. R. , Nunes D. S. , and Carvalho A. d. C. T. , Analgesic Activity of *Psychotria colorata* (Willd. ex R. & S.) Muell. Arg. Alkaloids, Journal of Ethnopharmacology. (1995) 48, no. 2, 77–83, 10.1016/0378-8741(95)01287-N, 2-s2.0-0028886515, 8583797. 8583797

[21] The Antibiotic Resistance Crisis: Part 1: Causes and Threats - PubMed, [cited 12 Mar 2025]. Available: https://pubmed.ncbi.nlm.nih.gov/25859123/.

[22] Mekonnen B. , Asrie A. B. , and Wubneh Z. B. , Antidiarrheal Activity of 80% Methanolic Leaf Extract of *Justicia schimperiana* , Evidence-Based Complementary and Alternative Medicine. (2018) 2018, 3037120, 10.1155/2018/3037120, 2-s2.0-85042544768, 29541140. 29541140 PMC5818970

[23] Kupchan S. M. , Fessler D. C. , Eakin M. A. , and Giacobbe T. J. , Reactions of Alpha Methylene Lactone Tumor Inhibitors With Model Biological Nucleophiles, Science. (1970) 168, no. 3929, 376–378, 10.1126/science.168.3929.376, 2-s2.0-0014952714, 5435896. 5435896

[24] Hossen M. S. , Refat K. M. N. H. , Faruque M. , Kundu P. , and Karmakar U. K. , Exploration of Antioxidative, Antidiarrheal, and Antihyperglycemic Properties of *Artocarpus chama* Leaves Along With In Silico Analysis, BioMed Research International. (2025) 2025, 9930195, 10.1155/bmri/9930195, 39949374. 39949374 PMC11824862

[25] Ezeja M. , Omeh Y. , Ezeigbo I. , and Ekechukwu A. , Evaluation of the Analgesic Activity of the Methanolic Stem Bark Extract of *Dialium guineense* (Wild), Annals of Medical and Health Sciences Research. (2011) 1, no. 1, 55–62, 23209955. 23209955 PMC3507093

[26] Jannat T. , Hossain M. J. , El-Shehawi A. M. , Kuddus M. R. , Rashid M. A. , Albogami S. , Jafri I. , El-Shazly M. , and Haque M. R. , Chemical and Pharmacological Profiling of *Wrightia coccinea* (Roxb. Ex Hornem.) Sims Focusing Antioxidant, Cytotoxic, Antidiarrheal, Hypoglycemic, and Analgesic Properties, Molecules. (2022) 27, no. 13, 10.3390/molecules27134024, 35807270. PMC9268577 35807270

[27] Tallei T. E. , Fatimawali , Adam A. A. , Elseehy M. M. , El-Shehawi A. M. , Mahmoud E. A. , Tania A. D. , Niode N. J. , Kusumawaty D. , Rahimah S. , Effendi Y. , Idroes R. , Celik I. , Hossain M. J. , and Emran T. B. , Fruit Bromelain-Derived Peptide Potentially Restrains the Attachment of SARS-CoV-2 Variants to hACE2: A Pharmacoinformatics Approach, Molecules. (2022) 27, no. 1, 10.3390/molecules27010260, 35011492. PMC8746556 35011492

[28] Khatun M. C. S. , Muhit M. A. , Hossain M. J. , Al-Mansur M. A. , and Rahman S. M. A. , Isolation of Phytochemical Constituents From *Stevia rebaudiana* (Bert.) and Evaluation of Their Anticancer, Antimicrobial and Antioxidant Properties via *In Vitro* and *In Silico* Approaches, Heliyon. (2021) 7, no. 12, e08475, 10.1016/j.heliyon.2021.e08475, 34917793. 34917793 PMC8645449

[29] Alam S. , Rashid M. A. , Sarker M. M. R. , Emon N. U. , Arman M. , Mohamed I. N. , and Haque M. R. , Antidiarrheal, Antimicrobial and Antioxidant Potentials of Methanol Extract of *Colocasia gigantea* Hook. f. Leaves: Evidenced From *In Vivo* and *In Vitro* Studies Along With Computer-Aided Approaches, BMC Complementary Medicine and Therapies. (2021) 21, no. 1, 10.1186/s12906-021-03290-6, 33845836. PMC8042880 33845836

[30] Mojica L. , Gonzalez de Mejia E. , Granados-Silvestre M. Á. , and Menjivar M. , Evaluation of the Hypoglycemic Potential of a Black Bean Hydrolyzed Protein Isolate and Its Pure Peptides Using *In Silico*, *In Vitro* and *In Vivo* Approaches, Journal of Functional Foods. (2017) 31, 274–286, 10.1016/j.jff.2017.02.006, 2-s2.0-85012098056.

[31] Ahmed T. , Khan A. U. , Abbass M. , Filho E. R. , Ud Din Z. , and Khan A. , Synthesis, Characterization, Molecular Docking, Analgesic, Antiplatelet and Anticoagulant Effects of Dibenzylidene Ketone Derivatives, Chemistry Central Journal. (2018) 12, no. 1, 10.1186/s13065-018-0507-1, 2-s2.0-85058054646, 30523436. PMC6768048 30523436

[32] Bikadi Z. and Hazai E. , Application of the PM6 Semi-Empirical Method to Modeling Proteins Enhances Docking Accuracy of AutoDock, Journal of Cheminformatics. (2009) 1, no. 1, 10.1186/1758-2946-1-15, 2-s2.0-74049093947, 20150996. PMC2820493 20150996

[33] Hasnat H. , Shompa S. A. , Richi F. T. , Islam M. M. , Suman M. H. , Ahmed N. U. , Ashrafi S. , Zaman A. , Saha T. , Islam M. A. , and Alam S. , Bioactive Secondary Metabolites to Combat Diabetic Complications: Evidenced From *In Silico* Study, Bangladesh Pharmaceutical Journal. (2023) 26, no. 2, 167–184, 10.3329/bpj.v26i2.67807.

[34] Jobaer M. A. , Ashrafi S. , Ahsan M. , Hasan C. M. , Rashid M. A. , Islam S. N. , and Masud M. M. , Phytochemical and Biological Investigation of an Indigenous Plant of Bangladesh, *Gynura procumbens* (Lour.) Merr.: Drug Discovery From Nature, Molecules. (2023) 28, no. 10, 10.3390/molecules28104186, 37241926. PMC10221986 37241926

[35] Islam M. , Sikder M. A. A. , Rashid M. A. , and Hossain M. K. , Phyto-Pharmacological Investigations of Leaves of *Cissus trifoliata* (L.), Dhaka University Journal of Pharmaceutical Sciences. (2022) 21, no. 1, 69–75, 10.3329/dujps.v21i1.60398.

[36] Hernández-Galicia E. , Calzada F. , Roman-Ramos R. , and Alarcón-Aguilar F. J. , Monoglycerides and Fatty Acids from *Ibervillea sonorae* Root: Isolation and Hypoglycemic Activity, Planta Medica. (2007) 73, no. 3, 236–240, 10.1055/s-2007-967117, 2-s2.0-34250311309. 17318782

[37] Ekins S. , Waller C. L. , Swaan P. W. , Cruciani G. , Wrighton S. A. , and Wikel J. H. , Progress in Predicting Human ADME Parameters In Silico, Journal of Pharmacological and Toxicological Methods. (2000) 44, no. 1, 251–272, 10.1016/S1056-8719(00)00109-X, 2-s2.0-0034460173. 11274894

[38] Prasanth D. S. N. B. K. , Murahari M. , Chandramohan V. , Panda S. P. , Atmakuri L. R. , and Guntupalli C. , *In Silico* Identification of Potential Inhibitors From *Cinnamon* Against Main Protease and Spike Glycoprotein of SARS CoV-2, Journal of Biomolecular Structure & Dynamics. (2021) 39, no. 13, 4618–4632, 10.1080/07391102.2020.1779129, 32567989. 32567989 PMC7332870

[39] Khan M. F. , Nahar N. , Bin R. R. , Chowdhury A. , and Rashid M. A. , Computational Investigations of Physicochemical, Pharmacokinetic, Toxicological Properties and Molecular Docking of Betulinic Acid, a Constituent of *Corypha taliera* (Roxb.) With Phospholipase A2 (PLA2), BMC Complementary and Alternative Medicine. (2018) 18, no. 1, 10.1186/s12906-018-2116-x, 2-s2.0-85041571305, 29391000. PMC5795847 29391000

[40] Lombardo F. , Gifford E. , and Shalaeva M. , In Silico ADME Prediction: Data, Models, Facts and Myths, Mini Reviews in Medicinal Chemistry. (2003) 3, no. 8, 861–875, 10.2174/1389557033487629, 2-s2.0-3042781869, 14529504. 14529504

[41] Kodidela S. , Pradhan S. C. , Muthukumaran J. , Dubashi B. , Santos-Silva T. , and Basu D. , Genotype Distribution of Dihydrofolatereductase Variants and Their Role in Disease Susceptibility to Acute Lymphoblastic Leukemia in Indian Population: An Experimental and Computational Analysis, Journal of Leukemia. (2016) 4, no. 1, 10.4172/2329-6917.1000209.

[42] Hwang J. , Youn K. , Ji Y. , Lee S. , Lim G. , Lee J. , Ho C. T. , Leem S. H. , and Jun M. , Biological and Computational Studies for Dual Cholinesterases Inhibitory Effect of Zerumbone, Nutrients. (2020) 12, no. 5, 10.3390/nu12051215, 32344943. PMC7281973 32344943

[43] Ma X. L. , Chen C. , and Yang J. , Predictive Model of Blood-Brain Barrier Penetration of Organic Compounds, Acta Pharmacologica Sinica. (2005) 26, no. 4, 500–512, 10.1111/j.1745-7254.2005.00068.x, 2-s2.0-16444373713, 15780201. 15780201

[44] Guttman Y. and Kerem Z. , Computer-Aided (In Silico) Modeling of Cytochrome P450-Mediated Food-Drug Interactions (FDI), International Journal of Molecular Sciences. (2022) 23, no. 15, 10.3390/ijms23158498, 35955630. PMC9369352 35955630

[45] El-Shamy N. T. , Alkaoud A. M. , Hussein R. K. , Ibrahim M. A. , Alhamzani A. G. , and Abou-Krisha M. M. , DFT, ADMET and Molecular Docking Investigations for the Antimicrobial Activity of 6,6′-Diamino-1,1′,3,3′-Tetramethyl-5,5′-(4-chlorobenzylidene)bis[pyrimidine-2,4(1H,3H)-dione], Molecules. (2022) 27, no. 3, 10.3390/molecules27030620, 35163880. PMC8839838 35163880

[46] Alves V. M. , Muratov E. N. , Zakharov A. , Muratov N. N. , Andrade C. H. , and Tropsha A. , Chemical Toxicity Prediction for Major Classes of Industrial Chemicals: Is It Possible to Develop Universal Models Covering Cosmetics, Drugs, and Pesticides?, Food and Chemical Toxicology. (2018) 112, 526–534, 10.1016/j.fct.2017.04.008, 2-s2.0-85017561579, 28412406. 28412406 PMC5638676

[47] Toropov A. A. , Toropova A. P. , Raska I. , Leszczynska D. , and Leszczynski J. , Comprehension of Drug Toxicity: Software and Databases, Computers in Biology and Medicine. (2014) 45, 20–25, 10.1016/j.compbiomed.2013.11.013, 2-s2.0-84890016373. 24480159

[48] Drwal M. N. , Banerjee P. , Dunkel M. , Wettig M. R. , and Preissner R. , ProTox: A Web Server for the *In Silico* Prediction of Rodent Oral Toxicity, Nucleic Acids Research. (2014) 42, no. W1, W53–W58, 10.1093/nar/gku401, 2-s2.0-84904818488, 24838562. 24838562 PMC4086068

[49] Banerjee P. , Eckert A. O. , Schrey A. K. , and Preissner R. , ProTox-II: A Webserver for the Prediction of Toxicity of Chemicals, Nucleic Acids Research. (2018) 46, no. W1, W257–W263, 10.1093/nar/gky318, 2-s2.0-85049470057, 29718510. 29718510 PMC6031011

[50] El-Din H. M. A. , Loutfy S. A. , Fathy N. , Elberry M. H. , Mayla A. M. , Kassem S. , and Naqvi A. , Molecular Docking Based Screening of Compounds Against VP40 From Ebola Virus, Bioinformation. (2016) 12, no. 3, 192–196, 10.6026/97320630012192, 28149054. 28149054 PMC5267963

[51] Lipinski C. A. , Lead- and Drug-Like Compounds: The Rule-of-Five Revolution, Drug Discovery Today: Technologies. (2004) 1, no. 4, 337–341, 10.1016/j.ddtec.2004.11.007, 2-s2.0-13244266921, 24981612. 24981612

[52] Tumilaar S. G. , Fatimawali F. , Niode N. J. , Effendi Y. , Idroes R. , Adam A. A. , Rakib A. , Emran T. B. , and Tallei T. E. , The Potential of Leaf Extract of Pangium edule Reinw as HIV-1 Protease Inhibitor: A Computational Biology Approach, Journal of Applied Pharmaceutical Science. (2020) 11, no. 1, 101–110, 10.7324/JAPS.2021.110112.

[53] Shaji D. , Molecular Docking Studies of Human MCT8 Protein With Soy Isoflavones in Allan-Herndon-Dudley Syndrome (AHDS), Journal of Pharmaceutical Analysis. (2018) 8, no. 5, 318–323, 10.1016/j.jpha.2018.07.001, 2-s2.0-85053689792, 30345146. 30345146 PMC6191963

[54] Tallei T. E. , Tumilaar S. G. , Niode N. J. , Fatimawali , Kepel B. J. , Idroes R. , Effendi Y. , Sakib S. A. , and Emran T. B. , Potential of Plant Bioactive Compounds as SARS-CoV-2 Main Protease (Mpro) and Spike (S) Glycoprotein Inhibitors: A Molecular Docking Study, Scientifica. (2020) 2020, 6307457, 10.1155/2020/6307457, 33425427. 33425427 PMC7773461

[55] Benet L. Z. , Hosey C. M. , Ursu O. , and Oprea T. I. , BDDCS, the Rule of 5 and Drugability, Advanced Drug Delivery Reviews. (2016) 101, 89–98, 10.1016/j.addr.2016.05.007, 2-s2.0-84973662249, 27182629. 27182629 PMC4910824

[56] Ashrafi S. , Rahman M. , Ahmed P. , Alam S. , and Hossain M. A. , Prospective Asian Plants With Corroborated Antiviral Potentials: Position Standing in Recent Years, Beni-Suef University Journal of Basic and Applied Sciences. (2022) 11, no. 1, 10.1186/s43088-022-00218-y, 35402627. PMC8980796 35402627

[57] Liu Y. , Shen Y. , Teng L. , Yang L. , Cao K. , Fu Q. , and Zhang J. , The Traditional Uses, Phytochemistry, and Pharmacology of *Stemona* Species: A Review, Journal of Ethnopharmacology. (2021) 265, 113112, 10.1016/j.jep.2020.113112, 32726680. 32726680

[58] Zhang Y. , Cai P. , Cheng G. , and Zhang Y. , A Brief Review of Phenolic Compounds Identified From Plants: Their Extraction, Analysis, and Biological Activity, Natural Product Communications. (2022) 17, no. 1, 10.1177/1934578X211069721.

[59] Villaño D. , Fernández-Pachón M. S. , Moyá M. L. , Troncoso A. M. , and García-Parrilla M. C. , Radical Scavenging Ability of Polyphenolic Compounds Towards DPPH Free Radical, Talanta. (2007) 71, no. 1, 230–235, 10.1016/j.talanta.2006.03.050, 2-s2.0-33845952418, 19071293. 19071293

[60] Santiago L. A. and Mayor A. B. R. , Lupeol: An Antioxidant Triterpene in *Ficus pseudopalma* Blanco (Moraceae), Asian Pacific Journal of Tropical Biomedicine. (2014) 4, no. 2, 109–118, 10.1016/S2221-1691(14)60218-5, 2-s2.0-84899570175, 25182281. 25182281 PMC3819478

[61] Renuka Devi R. and Arumughan C. , Antiradical Efficacy of Phytochemical Extracts From Defatted Rice Bran, Food and Chemical Toxicology. (2007) 45, no. 10, 2014–2021, 10.1016/j.fct.2007.04.020, 2-s2.0-34547821931, 17574716. 17574716

[62] Jain T. , Singh M. P. , Bhardwaj H. , and Gohil K. J. , Review on Pharmacology Activities of *Justicia gendarussa Burm F*., Pharmacological Research-Modern Chinese Medicine. (2024) 10, 100339, 10.1016/j.prmcm.2023.100339.

[63] Mondal M. , Hossain M. M. , Rahman M. A. , Saha S. , Uddin N. , Hasan M. R. , Kader A. , Wahed T. B. , Kundu S. K. , Islam M. T. , and Mubarak M. S. , Hepatoprotective and Antioxidant Activities of *Justicia gendarussa* Leaf Extract in Carbofuran-Induced Hepatic Damage in Rats, Chemical Research in Toxicology. (2019) 32, no. 12, 2499–2508, 10.1021/acs.chemrestox.9b00345, 31696704. 31696704

[64] Sudhahar V. , Kumar S. A. , and Varalakshmi P. , Role of Lupeol and Lupeol Linoleate on Lipemic-Oxidative Stress in Experimental Hypercholesterolemia, Life Sciences. (2006) 78, no. 12, 1329–1335, 10.1016/j.lfs.2005.07.011, 2-s2.0-32044454032, 16216277. 16216277

[65] Gupta R. , Sharma A. K. , Dobhal M. P. , Sharma M. C. , and Gupta R. S. , Antidiabetic and Antioxidant Potential of *β*-Sitosterol in Streptozotocin-Induced Experimental Hyperglycemia, Journal of Diabetes. (2011) 3, no. 1, 29–37, 10.1111/j.1753-0407.2010.00107.x, 2-s2.0-79951781220, 21143769. 21143769

[66] Hidalgo F. J. , Mercedes León M. , and Zamora R. , Effect of *β*-Sitosterol in the Antioxidative Activity of Oxidized Lipid–Amine Reaction Products, Food Research International. (2009) 42, no. 8, 1215–1222, 10.1016/j.foodres.2009.06.001, 2-s2.0-67651004681.

[67] Zulfiker A. H. M. , Rahman M. M. , Hossain M. K. , Hamid K. , Mazumder M. E. H. , and Rana M. S. , In Vivo Analgesic Activity of Ethanolic Extracts of Two Medicinal Plants - *Scoparia dulcis* L. and *Ficus racemosa* Linn, Biologie et Médecine. (2010) 2, no. 2, 42–48.

[68] Shreedhara C. , Vaidya V. , Vagdevi H. , Latha K. , Muralikrishna K. , and Krupanidhi A. , Screening of *Bauhinia purpurea* Linn. for Analgesic and Anti-Inflammatory Activities, Indian Journal of Pharmacology. (2009) 41, no. 2, 75–79, 10.4103/0253-7613.51345, 2-s2.0-67649878228, 20336222. 20336222 PMC2841237

[69] Jothimanivannan C. , Kumar R. , and Subramanian N. , Anti-Inflammatory and Analgesic Activities of Ethanol Extract of Aerial Parts of *Justicia gendarussa* Burm, International Journal of Pharmacology. (2010) 6, no. 3, 278–283, 10.3923/ijp.2010.278.283.

[70] Nirmal S. A. , Pal Subodh C. , Subhash C. M. , and Anuja N. P. , Analgesic and Anti-Inflammatory Activity of *β*-Sitosterol Isolated From *Nyctanthes arbortristis* Leaves, Inflammopharmacology. (2012) 20, no. 4, 219–224, 10.1007/s10787-011-0110-8, 2-s2.0-84864949899, 22207496. 22207496

[71] Gidudu J. , Sack D. A. , Pina M. , Hudson M. J. , Kohl K. S. , Bishop P. , Chatterjee A. , Chiappini E. , Compingbutra A. , da Costa C. , Fernandopulle R. , Fischer T. K. , Haber P. , Masana W. , de Menezes M. R. , Kang G. , Khuri-Bulos N. , Killion L. A. , Nair C. , Poerschke G. , Rath B. , Salazar-Lindo E. , Setse R. , Wenger P. , Wong V. C. , Zaman K. , and Brighton Collaboration Diarrhea Working Group , Diarrhea: Case Definition and Guidelines for Collection, Analysis, and Presentation of Immunization Safety Data, Vaccine. (2011) 29, no. 5, 1053–1071, 10.1016/j.vaccine.2010.11.065, 2-s2.0-78751584989, 21130754. 21130754

[72] Zewdie K. A. , Bhoumik D. , Wondafrash D. Z. , and Tuem K. B. , Evaluation of In-Vivo Antidiarrhoeal and In-Vitro Antibacterial Activities of the Root Extract of *Brucea antidysenterica* J. F. Mill (Simaroubaceae), BMC Complementary Medicine and Therapies. (2020) 20, no. 1, 10.1186/s12906-020-03001-7, 32605618. PMC7325256 32605618

[73] Rahman S. M. M. , Saha B. , Rahman M. H. , Islam A. , Nahar S. , Hasan M. T. I. , and Sakib M. N. , Phytochemical Screening, Acute Toxicity, Antinociceptive and Antidiarrheal Activity of *Gendarussa vulgaris* Leaves Extract, Journal of Pharmacognosy and Phytochemistry. (2018) 7, no. 5, 577–584, Available: https://www.phytojournal.com/archives/2018.v7.i5.5603/phytochemical-screening-acute-toxicity-antinociceptive-and-antidiarrheal-activity-of-ltemgtgendarussa-vulgarisltemgt-leaves-extract.

[74] Wang J. B. , Qin Y. , Kong W. J. , Wang Z. W. , Zeng L. N. , Fang F. , Jin C. , Zhao Y. L. , and Xiao X. H. , Identification of the Antidiarrhoeal Components in Official Rhubarb Using Liquid Chromatography-Tandem Mass Spectrometry, Food Chemistry. (2011) 129, no. 4, 1737–1743, 10.1016/j.foodchem.2011.06.041, 2-s2.0-80051786155.

[75] Yao W. R. , Wang H. Y. , Wang S. T. , Sun S. L. , Zhou J. , and Luan Y. Y. , Assessment of the Antibacterial Activity and the Antidiarrheal Function of Flavonoids From Bayberry Fruit, Journal of Agricultural and Food Chemistry. (2011) 59, no. 10, 5312–5317, 10.1021/jf200211m, 2-s2.0-79957964234, 21469731. 21469731

[76] Wang Y. , Campbell T. , Perry B. , Beaurepaire C. , and Qin L. , Hypoglycemic and Insulin-Sensitizing Effects of Berberine in High-Fat Diet- and Streptozotocin-Induced Diabetic Rats, Metabolism. (2011) 60, no. 2, 298–305, 10.1016/j.metabol.2010.02.005, 2-s2.0-78751647742, 20304443. 20304443

[77] Jouad H. , Haloui M. , Rhiouani H. , El Hilaly J. , and Eddouks M. , Ethnobotanical Survey of Medicinal Plants Used for the Treatment of Diabetes, Cardiac and Renal Diseases in the North Centre Region of Morocco (Fez-Boulemane), Journal of Ethnopharmacology. (2001) 77, no. 2-3, 175–182, 10.1016/S0378-8741(01)00289-6, 2-s2.0-0034891429, 11535361. 11535361

[78] Ponnulakshmi R. , Shyamaladevi B. , Vijayalakshmi P. , and Selvaraj J. , *In Silico* and *In Vivo* Analysis to Identify the Antidiabetic Activity of Beta Sitosterol in Adipose Tissue of High Fat Diet and Sucrose Induced Type-2 Diabetic Experimental Rats, Toxicology Mechanisms and Methods. (2019) 29, no. 4, 276–290, 10.1080/15376516.2018.1545815, 2-s2.0-85060173080, 30461321. 30461321

[79] Nascimento G. G. F. , Locatelli J. , Freitas P. C. , and Silva G. L. , Antibacterial Activity of Plant Extracts and Phytochemicals on Antibiotic-Resistant Bacteria, Brazilian Journal of Microbiology. (2000) 31, no. 4, 247–256, 10.1590/S1517-83822000000400003, 2-s2.0-0013249285.

[80] Vital P. G. and Rivera W. , Antimicrobial Activity and Cytotoxicity of *Chromolaena odorata* (L. f.) King and Robinson and *Uncaria perrottetii* (A. Rich) Merr. Extracts, Journal of Medicinal Plants Research. (2009) 3, no. 7, 511–518.

[81] Nirmalraj S. , Ravikumar M. , Mahendrakumar M. , Bharath B. , and Perinbam K. , Antibacterial and Anti-Inflammatory Activity of *Justicia gendarussa* Burm. F. Leaves, Journal of Plant Sciences. (2015) 10, no. 2, 70–74.

[82] Sikder M. A. A. , Hossian A. K. M. N. , Siddique A. B. , Ahmed M. , Kaisar M. A. , and Rashid M. A. , *In Vitro* Antimicrobial Screening of Four Reputed Bangladeshi Medicinal Plants, Polymer Journal. (2011) 3, no. 24, 72–76, 10.5530/PJ.2011.24.14, 2-s2.0-80053214159.

[83] Ali M. F. , Mahmud S. , Mohiuddin R. B. , Chowdhury K. , and Mohiuddin A. K. , Screening of Preliminary Phytochemicals, Molecular Identification, and Antimicrobial and Anti-Inflammatory Activity of *Justicia gendarussa* , Evidence-Based Complementary and Alternative Medicine. (2023) 2023, 6885353, 10.1155/2023/6885353.

[84] Subramanian N. , Jothimanivannan C. , and Moorthy K. , Antimicrobial Activity and Preliminary Phytochemical Screening of *Justicia gendarussa* (Burm. f.) Against Human Pathogens, Asian Journal of Pharmaceutical and Clinical Research. (2012) 5, no. 3, 229–233.

[85] Rahman A. U. , Abdullah A. , Faisal S. , Mansour B. , and Yahya G. , Unlocking the Therapeutic Potential of *Nigella sativa* Extract: Phytochemical Analysis and Revealing Antimicrobial and Antioxidant Marvels, BMC Complementary Medicine and Therapies. (2024) 24, no. 1, 10.1186/s12906-024-04470-w, 38997638. PMC11241953 38997638

