# Supplementary material for: Phenolic Compounds of Justicia gendarussa Show Pharmacological Potentials Against Pain, Oxidation, Hyperglycemia, Diarrhea, and Microbes: Phytopharmacological and Computational Approaches
Source: Biomed Res Int. 2025 Dec 9;2025:2561508. doi: 10.1155/bmri/2561508 (PMC12686832; doi:10.1155/bmri/2561508)
Supplement: Supplementary file 1 — Supporting Information Additional supporting information can be found online in the Supporting Information section. [file BMRI-2025-2561508-s001.docx]

Article type: Original Research

Phenolic compounds of *Justicia gendarussa* show pharmacological potentials against pain, oxidation, hyperglycemia, diarrhea, and microbes: Phyto-pharmacological and computational approaches

**Authors:**

Farzana Akter Munny^1ψ^, Mehedi Islam^2ψ^, Mahafuza Akter^3^, Mushtahsin Ferdousi^2^, Md. Moaz Ahmed Asif^4^, Md. Solaiman Hossain^5^, Sabrina Sharmin^2^, Md Zahidul Islam^6^, Md. Aslam Hossain^1*^, Md. Rabiul Islam^2*^

*^ψ^ These authors contributed equally to this work and the names are arranged in alphabetic order.*

**Affiliations:**

^1^Department of Pharmaceutical Chemistry, Faculty of Pharmacy, University of Dhaka, Dhaka 1000, Bangladesh

^2^School of Pharmacy, BRAC University, Kha 224 Bir Uttam Rafiqul Islam Avenue, Merul Badda, Dhaka 1212, Bangladesh

^3^Department of Pharmaceutical Technology and Biopharmaceutics, School of Health and Life Sciences, North South University, 74/A, Bashundhara, Dhaka 1229, Bangladesh

^4^Department of Pharmacy, Faculty of Science and Engineering, University of Information Technology and Sciences, Road 5, Block J, Baridhara, Naya Nagar Road, Dhaka 1212, Bangladesh

^5^Department of Pharmacy, School of Pharmaceutical Sciences, State University of Bangladesh, 696 Kendua, Kanchan, Rupganj, Narayanganj, Dhaka 1461, Bangladesh

^6^Deapartment of Biotechnology and Genetic Engineering, Jahangirnagar University, Savar, Dhaka-1342, Bangladesh

**Author’s Email:**

Farzana Akter Munny: [farzanamunny2@gmail.com](mailto:farzanamunny2@gmail.com); Mehedi Islam: [mehediislam1294@gmail.com](mailto:mehediislam1294@gmail.com); Mahafuza Akter: [mahafuza.sub@gmail.com](mailto:mahafuza.sub@gmail.com); Mushtahsin Ferdousi: [mushtahsin1996@gmail.com](mailto:mushtahsin1996@gmail.com); Md. Moaz Ahmed Asif: [moaz.ahmed@uits.edu.bd](mailto:moaz.ahmed@uits.edu.bd)

Md. Solaiman Hossain: [solaiman.pharmacy@sub.edu.bd](mailto:solaiman.pharmacy@sub.edu.bd); Sabrina Sharmin: [sabrina.sharmin@bracu.ac.bd](mailto:sabrina.sharmin@bracu.ac.bd); Md. Zahidul Islam: [zahidul@juniv.edu](mailto:zahidul@juniv.edu); Md. Aslam Hossain: [aslamsaga@du.ac.bd](mailto:aslamsaga@du.ac.bd); Md. Rabiul Islam: [robi.ayaan@gmail.com](mailto:robi.ayaan@gmail.com)

**^*^Correspondence:**

**Md. Aslam Hossain, PhD**

Professor, Department of Pharmaceutical Chemistry, Faculty of Pharmacy, University of Dhaka, Dhaka 1000, Bangladesh. Mobile (Personal): +88-01711059401; Email: [aslamsaga@du.ac.bd](mailto:aslamsaga@du.ac.bd)

**Md. Rabiul Islam, PhD**

School of Pharmacy, BRAC University, Kha 224 Bir Uttam Rafiqul Islam Avenue, Merul Badda, Dhaka 1212, Bangladesh. Mobile (Personal): +88 01916031831

Email: [robi.ayaan@gmail.com](mailto:robi.ayaan@gmail.com); ORCID: <https://orcid.org/0000-0003-2820-3144>

**Table S1.** Interactions of amino acids of the glutathione reductase enzyme (PDB ID: 3GRS) with the selected compounds, Lupeol (**C1**), β-sitosterol (**C2**), and 1-monostearin (**C3**) from *Justica gendarussa* during molecular docking.

| **Compound** | **Bond (AA & Ligand)** | **Bond Length** | **Bond Type** | **Bond Nature** |
| --- | --- | --- | --- | --- |
| **C1** | A:HIS52:HN - A:GLU50:OE2 | 1.76445 | Hydrogen Bond | Conventional Hydrogen Bond |
|  | A:VAL61:HN - A:THR57:O | 1.92668 | Hydrogen Bond | Conventional Hydrogen Bond |
|  | A:THR156:HG1 - A:ASN294:O | 2.1502 | Hydrogen Bond | Conventional Hydrogen Bond |
|  | A:ASN294:HN - A:GLY157:O | 2.01594 | Hydrogen Bond | Conventional Hydrogen Bond |
|  | A:ASN294:HD22 - N:UNK1:O | 2.61675 | Hydrogen Bond | Conventional Hydrogen Bond |
|  | A:VAL61 - N:UNK1 | 4.85713 | Hydrophobic | Alkyl |
|  | N:UNK1:C - A:VAL61 | 4.52631 | Hydrophobic | Alkyl |
|  | A:HIS52 - N:UNK1 | 4.45938 | Hydrophobic | Pi-Alkyl |
|  | A:HIS52 - N:UNK1 | 4.98179 | Hydrophobic | Pi-Alkyl |
|  | A:HIS52 - N:UNK1:C | 4.81531 | Hydrophobic | Pi-Alkyl |
| **C2** | N:UNK1:H - A:GLN182:OE1 | 2.73758 | Hydrogen Bond | Conventional Hydrogen Bond |
|  | A:LYS53 - N:UNK1 | 5.34456 | Hydrophobic | Alkyl |
|  | A:VAL61 - N:UNK1 | 5.08095 | Hydrophobic | Alkyl |
|  | A:VAL61 - N:UNK1 | 4.89343 | Hydrophobic | Alkyl |
|  | A:VAL61 - N:UNK1 | 4.44561 | Hydrophobic | Alkyl |
| **C3** | A:CYS58:HN - N:UNK1:O | 2.37275 | Hydrogen Bond | Conventional Hydrogen Bond |
|  | N:UNK1:H - A:SER30:OG | 2.26763 | Hydrogen Bond | Conventional Hydrogen Bond |
|  | A:GLY157:CA - N:UNK1:O | 3.7828 | Hydrogen Bond | Carbon Hydrogen Bond |
|  | N:UNK1:C - A:THR57:OG1 | 3.77036 | Hydrogen Bond | Carbon Hydrogen Bond |
|  | A:LYS53 - N:UNK1 | 4.26456 | Hydrophobic | Alkyl |
|  | A:LYS53 - N:UNK1 | 5.37217 | Hydrophobic | Alkyl |
|  | N:UNK1:C - A:VAL61 | 4.53992 | Hydrophobic | Alkyl |
|  | A:HIS52 - N:UNK1 | 4.70976 | Hydrophobic | Pi-Alkyl |
|  | A:HIS52 - N:UNK1 | 4.97644 | Hydrophobic | Pi-Alkyl |

**Table S2.** Interactions of amino acids of the mu-opioid receptor enzyme (PDB ID: 5C1M) with the selected compounds, Lupeol (**C1**), β-sitosterol (**C2**), and 1-monostearin (**C3**) from *Justica gendarussa* during molecular docking.

| **Compound** | **Bond (AA & Ligand)** | **Bond Length** | **Bond Type** | **Bond Nature** |
| --- | --- | --- | --- | --- |
| **C1** | N:UNK1:C - A:TYR148 | 3.9155 | Hydrophobic | Pi-Sigma |
|  | A:VAL143 - N:UNK1 | 5.0941 | Hydrophobic | Alkyl |
|  | A:ILE144 - N:UNK1 | 4.57675 | Hydrophobic | Alkyl |
|  | A:MET151 - N:UNK1 | 5.14353 | Hydrophobic | Alkyl |
|  | N:UNK1 - A:MET151 | 5.29084 | Hydrophobic | Alkyl |
|  | N:UNK1 - A:ILE296 | 3.74144 | Hydrophobic | Alkyl |
|  | N:UNK1:C - A:ILE296 | 5.06483 | Hydrophobic | Alkyl |
|  | N:UNK1:C - A:VAL300 | 4.02424 | Hydrophobic | Alkyl |
|  | N:UNK1:C - A:VAL143 | 4.04143 | Hydrophobic | Alkyl |
|  | N:UNK1:C - A:ILE144 | 3.44654 | Hydrophobic | Alkyl |
|  | N:UNK1:C - A:VAL143 | 5.09743 | Hydrophobic | Alkyl |
|  | N:UNK1:C - A:ILE144 | 3.50012 | Hydrophobic | Alkyl |
|  | N:UNK1:C - A:CYS217 | 4.49665 | Hydrophobic | Alkyl |
|  | N:UNK1:C - A:ILE296 | 2.92488 | Hydrophobic | Alkyl |
|  | N:UNK1:C - A:ILE322 | 4.56811 | Hydrophobic | Alkyl |
|  | N:UNK1:C - A:MET151 | 4.74672 | Hydrophobic | Alkyl |
|  | N:UNK1:C - A:ILE296 | 4.75986 | Hydrophobic | Alkyl |
| **C2** | A:TYR148 - N:UNK1 | 5.35421 | Hydrophobic | Pi-Alkyl |
|  | A:TRP293 - N:UNK1 | 5.387 | Hydrophobic | Pi-Alkyl |
|  | A:TRP293 - N:UNK1:C | 5.18053 | Hydrophobic | Pi-Alkyl |
|  | A:TRP293 - N:UNK1:C | 3.81944 | Hydrophobic | Pi-Alkyl |
|  | A:HIS297 - N:UNK1 | 4.66786 | Hydrophobic | Pi-Alkyl |
|  | A:TYR326 - N:UNK1:C | 5.43353 | Hydrophobic | Pi-Alkyl |
|  | A:TYR326 - N:UNK1:C | 4.825 | Hydrophobic | Pi-Alkyl |
|  | N:UNK1:C - A:MET151 | 4.79941 | Hydrophobic | Alkyl |
|  | N:UNK1:C - A:VAL236 | 4.0895 | Hydrophobic | Alkyl |
|  | N:UNK1:C - A:ILE322 | 4.00017 | Hydrophobic | Alkyl |
|  | N:UNK1:C - A:VAL143 | 5.01487 | Hydrophobic | Alkyl |
|  | N:UNK1:C - A:ILE144 | 4.77507 | Hydrophobic | Alkyl |
|  | N:UNK1:C - A:ILE144 | 3.56563 | Hydrophobic | Alkyl |
|  | A:TRP133 - N:UNK1:C | 5.24243 | Hydrophobic | Pi-Alkyl |
|  | A:TYR148 - N:UNK1 | 5.32835 | Hydrophobic | Pi-Alkyl |
|  | A:HIS297 - N:UNK1 | 3.89139 | Hydrophobic | Pi-Alkyl |
|  | A:HIS297 - N:UNK1:C | 3.97662 | Hydrophobic | Pi-Alkyl |
|  | A:TYR326 - N:UNK1:C | 5.38836 | Hydrophobic | Pi-Alkyl |
| **C3** | N:UNK1:H - A:ASP147:OD2 | 2.62928 | Hydrogen Bond | Conventional Hydrogen Bond |
|  | N:UNK1:H - A:TYR326:OH | 2.93988 | Hydrogen Bond | Conventional Hydrogen Bond |
|  | A:HIS54:CD2 - N:UNK1:O | 3.53224 | Hydrogen Bond | Carbon Hydrogen Bond |
|  | N:UNK1:C - A:HIS54:NE2 | 3.68092 | Hydrogen Bond | Carbon Hydrogen Bond |
|  | N:UNK1:C - A:HIS54 | 3.63108 | Hydrophobic | Pi-Sigma |
|  | A:VAL236 - N:UNK1 | 4.48205 | Hydrophobic | Alkyl |
|  | A:VAL300 - N:UNK1 | 4.65458 | Hydrophobic | Alkyl |
|  | N:UNK1 - A:ILE296 | 4.97044 | Hydrophobic | Alkyl |
|  | N:UNK1 - A:ILE322 | 4.46265 | Hydrophobic | Alkyl |
|  | N:UNK1 - A:ILE296 | 4.2701 | Hydrophobic | Alkyl |
|  | N:UNK1 - A:ILE322 | 4.25767 | Hydrophobic | Alkyl |
|  | N:UNK1 - A:MET151 | 4.71944 | Hydrophobic | Alkyl |
|  | N:UNK1 - A:ILE296 | 4.7453 | Hydrophobic | Alkyl |
|  | N:UNK1:C - A:LEU232 | 4.09006 | Hydrophobic | Alkyl |
|  | N:UNK1:C - A:LYS233 | 4.04704 | Hydrophobic | Alkyl |
|  | N:UNK1:C - A:VAL236 | 4.23308 | Hydrophobic | Alkyl |
|  | A:TYR148 - N:UNK1 | 5.38672 | Hydrophobic | Pi-Alkyl |
|  | A:TRP318 - N:UNK1 | 5.19219 | Hydrophobic | Pi-Alkyl |
|  | A:TYR326 - N:UNK1 | 4.7878 | Hydrophobic | Pi-Alkyl |

**Table S3.** Interactions of amino acids of the Cox-2 inhibitor (PDB ID: 1CX2) with the selected compounds, Lupeol (**C1**), β-sitosterol (**C2**), and 1-monostearin (**C3**) from *Justica gendarussa* during molecular docking.

| **Compound** | **Bond (AA & Ligand)** | **Bond Length** | **Bond Type** | **Bond Nature** |
| --- | --- | --- | --- | --- |
| **C1** | A:VAL116 - N:UNK1 | 4.97588 | Hydrophobic | Alkyl |
|  | A:VAL349 - N:UNK1 | 4.57724 | Hydrophobic | Alkyl |
|  | A:VAL349 - N:UNK1 | 5.05598 | Hydrophobic | Alkyl |
|  | A:VAL349 - N:UNK1 | 4.40164 | Hydrophobic | Alkyl |
|  | A:LEU352 - N:UNK1 | 4.66103 | Hydrophobic | Alkyl |
|  | A:VAL523 - N:UNK1 | 4.0528 | Hydrophobic | Alkyl |
|  | A:ALA527 - N:UNK1 | 3.5004 | Hydrophobic | Alkyl |
|  | A:ALA527 - N:UNK1 | 4.51323 | Hydrophobic | Alkyl |
|  | A:ALA527 - N:UNK1 | 4.88061 | Hydrophobic | Alkyl |
|  | N:UNK1 - A:LEU352 | 5.03615 | Hydrophobic | Alkyl |
|  | N:UNK1 - A:MET522 | 5.42457 | Hydrophobic | Alkyl |
|  | N:UNK1 - A:LEU359 | 4.32371 | Hydrophobic | Alkyl |
|  | N:UNK1 - A:LEU531 | 4.49715 | Hydrophobic | Alkyl |
|  | N:UNK1:C - A:MET522 | 5.32233 | Hydrophobic | Alkyl |
|  | N:UNK1:C - A:VAL523 | 3.84957 | Hydrophobic | Alkyl |
|  | N:UNK1:C - A:VAL116 | 4.20347 | Hydrophobic | Alkyl |
|  | N:UNK1:C - A:LEU359 | 3.36714 | Hydrophobic | Alkyl |
|  | N:UNK1:C - A:VAL116 | 3.05068 | Hydrophobic | Alkyl |
|  | N:UNK1:C - A:LEU352 | 4.51196 | Hydrophobic | Alkyl |
|  | A:TYR348 - N:UNK1:C | 4.77048 | Hydrophobic | Pi-Alkyl |
|  | A:TYR355 - N:UNK1 | 5.29366 | Hydrophobic | Pi-Alkyl |
|  | A:TYR355 - N:UNK1:C | 4.19177 | Hydrophobic | Pi-Alkyl |
|  | A:PHE381 - N:UNK1:C | 4.52736 | Hydrophobic | Pi-Alkyl |
|  | A:TYR385 - N:UNK1 | 5.24068 | Hydrophobic | Pi-Alkyl |
|  | A:TYR385 - N:UNK1:C | 4.86084 | Hydrophobic | Pi-Alkyl |
|  | A:TYR385 - N:UNK1:C | 4.01483 | Hydrophobic | Pi-Alkyl |
|  | A:TRP387 - N:UNK1 | 4.5841 | Hydrophobic | Pi-Alkyl |
|  | A:TRP387 - N:UNK1:C | 5.16483 | Hydrophobic | Pi-Alkyl |
|  | A:PHE518 - N:UNK1 | 5.0995 | Hydrophobic | Pi-Alkyl |
|  | A:PHE518 - N:UNK1 | 5.03048 | Hydrophobic | Pi-Alkyl |
| **C2** | A:VAL116 - N:UNK1 | 5.41325 | Hydrophobic | Alkyl |
|  | A:VAL349 - N:UNK1 | 5.05758 | Hydrophobic | Alkyl |
|  | A:VAL349 - N:UNK1 | 5.24949 | Hydrophobic | Alkyl |
|  | A:LEU352 - N:UNK1 | 5.23093 | Hydrophobic | Alkyl |
|  | A:LEU352 - N:UNK1 | 5.06412 | Hydrophobic | Alkyl |
|  | A:VAL523 - N:UNK1 | 4.61978 | Hydrophobic | Alkyl |
|  | A:VAL523 - N:UNK1 | 5.48768 | Hydrophobic | Alkyl |
|  | A:VAL523 - N:UNK1 | 4.61993 | Hydrophobic | Alkyl |
|  | A:ALA527 - N:UNK1 | 4.81548 | Hydrophobic | Alkyl |
|  | A:ALA527 - N:UNK1 | 3.98712 | Hydrophobic | Alkyl |
|  | N:UNK1:C - A:LEU352 | 4.03234 | Hydrophobic | Alkyl |
|  | N:UNK1 - A:LEU359 | 4.65715 | Hydrophobic | Alkyl |
|  | N:UNK1:C - A:VAL116 | 4.79215 | Hydrophobic | Alkyl |
|  | N:UNK1:C - A:LEU531 | 3.59083 | Hydrophobic | Alkyl |
|  | N:UNK1:C - A:MET113 | 5.13432 | Hydrophobic | Alkyl |
|  | N:UNK1:C - A:VAL116 | 4.52083 | Hydrophobic | Alkyl |
|  | N:UNK1:C - A:VAL349 | 4.92989 | Hydrophobic | Alkyl |
|  | N:UNK1:C - A:LEU359 | 3.70963 | Hydrophobic | Alkyl |
|  | N:UNK1:C - A:LEU531 | 4.7171 | Hydrophobic | Alkyl |
|  | A:TYR355 - N:UNK1 | 4.23494 | Hydrophobic | Pi-Alkyl |
|  | A:PHE381 - N:UNK1 | 5.29382 | Hydrophobic | Pi-Alkyl |
|  | A:TYR385 - N:UNK1 | 4.55311 | Hydrophobic | Pi-Alkyl |
|  | A:TYR385 - N:UNK1:C | 5.37724 | Hydrophobic | Pi-Alkyl |
|  | A:TRP387 - N:UNK1 | 5.30872 | Hydrophobic | Pi-Alkyl |
|  | A:TRP387 - N:UNK1:C | 4.98784 | Hydrophobic | Pi-Alkyl |
|  | A:PHE518 - N:UNK1 | 4.83111 | Hydrophobic | Pi-Alkyl |
| **C3** | A:GLN192:HE21 - N:UNK1:O | 2.34492 | Hydrogen Bond | Conventional Hydrogen Bond |
|  | N:UNK1:H - A:LEU352:O | 2.50891 | Hydrogen Bond | Conventional Hydrogen Bond |
|  | N:UNK1:H - A:SER353:O | 1.94402 | Hydrogen Bond | Conventional Hydrogen Bond |
|  | N:UNK1:C - A:HIS90:NE2 | 3.37236 | Hydrogen Bond | Carbon Hydrogen Bond |
|  | N:UNK1:C - A:SER353:O | 3.58263 | Hydrogen Bond | Carbon Hydrogen Bond |
|  | A:VAL349 - N:UNK1 | 3.9941 | Hydrophobic | Alkyl |
|  | A:VAL349 - N:UNK1 | 4.41567 | Hydrophobic | Alkyl |
|  | A:VAL523 - N:UNK1 | 4.31243 | Hydrophobic | Alkyl |
|  | A:ALA527 - N:UNK1 | 4.90183 | Hydrophobic | Alkyl |
|  | A:ALA527 - N:UNK1 | 4.77606 | Hydrophobic | Alkyl |
|  | A:ALA527 - N:UNK1 | 3.83049 | Hydrophobic | Alkyl |
|  | N:UNK1 - A:LEU352 | 5.42231 | Hydrophobic | Alkyl |
|  | N:UNK1 - A:LEU531 | 4.72253 | Hydrophobic | Alkyl |
|  | N:UNK1:C - A:VAL116 | 4.0945 | Hydrophobic | Alkyl |
|  | N:UNK1:C - A:VAL349 | 5.24192 | Hydrophobic | Alkyl |
|  | N:UNK1:C - A:LEU359 | 4.23822 | Hydrophobic | Alkyl |
|  | N:UNK1:C - A:LEU531 | 4.8748 | Hydrophobic | Alkyl |
|  | A:TYR385 - N:UNK1 | 4.81928 | Hydrophobic | Pi-Alkyl |
|  | A:TRP387 - N:UNK1 | 5.31694 | Hydrophobic | Pi-Alkyl |
|  | A:PHE518 - N:UNK1 | 5.06472 | Hydrophobic | Pi-Alkyl |

**Table S4.** Interactions of amino acids of the kappa opioid receptor enzyme (PDB ID: 6VI4) with the selected compounds, Lupeol (**C1**), β-sitosterol (**C2**), and 1-monostearin (**C3**) from *Justica gendarussa* during molecular docking.

| **Compound** | **Bond (AA & Ligand)** | **Bond Length** | **Bond Type** | **Bond Nature** |
| --- | --- | --- | --- | --- |
| **C1** | N:UNK1 - B:ILE180 | 4.22427 | Hydrophobic | Alkyl |
|  | N:UNK1:C - B:ILE180 | 4.26448 | Hydrophobic | Alkyl |
|  | N:UNK1:C - B:LEU184 | 4.97809 | Hydrophobic | Alkyl |
|  | N:UNK1:C - B:LEU103 | 5.02633 | Hydrophobic | Alkyl |
|  | N:UNK1:C - B:LEU103 | 4.77751 | Hydrophobic | Alkyl |
|  | B:TYR140 - N:UNK1:C | 4.83837 | Hydrophobic | Pi-Alkyl |
|  | B:TRP183 - N:UNK1 | 4.62276 | Hydrophobic | Pi-Alkyl |
|  | B:TRP183 - N:UNK1:C | 4.21624 | Hydrophobic | Pi-Alkyl |
|  | B:TRP183 - N:UNK1 | 4.71069 | Hydrophobic | Pi-Alkyl |
| **C2** | N:UNK1:C - B:TRP183 | 3.72955 | Hydrophobic | Pi-Sigma |
|  | N:UNK1:C - B:TRP183 | 3.62286 | Hydrophobic | Pi-Sigma |
|  | B:LEU103 - N:UNK1 | 5.3926 | Hydrophobic | Alkyl |
|  | N:UNK1:C - B:LEU107 | 4.34477 | Hydrophobic | Alkyl |
|  | N:UNK1:C - B:ILE137 | 4.4346 | Hydrophobic | Alkyl |
|  | N:UNK1:C - B:ILE133 | 4.27067 | Hydrophobic | Alkyl |
|  | N:UNK1:C - B:ILE137 | 5.40841 | Hydrophobic | Alkyl |
|  | B:TYR140 - N:UNK1 | 5.14406 | Hydrophobic | Pi-Alkyl |
|  | B:TYR140 - N:UNK1:C | 4.58503 | Hydrophobic | Pi-Alkyl |
|  | B:TRP183 - N:UNK1 | 4.35614 | Hydrophobic | Pi-Alkyl |
|  | B:TRP183 - N:UNK1 | 4.70786 | Hydrophobic | Pi-Alkyl |
|  | B:TRP183 - N:UNK1 | 4.48366 | Hydrophobic | Pi-Alkyl |
|  | B:TRP183 - N:UNK1 | 5.25286 | Hydrophobic | Pi-Alkyl |
|  | B:TRP183 - N:UNK1:C | 5.46909 | Hydrophobic | Pi-Alkyl |
| **C3** | N:UNK1:C - B:TRP183 | 3.70174 | Hydrophobic | Pi-Sigma |
|  | N:UNK1 - B:ILE180 | 4.44466 | Hydrophobic | Alkyl |
|  | N:UNK1:C - B:LEU103 | 4.86131 | Hydrophobic | Alkyl |
|  | B:PHE99 - N:UNK1:C | 5.30218 | Hydrophobic | Pi-Alkyl |
|  | B:TRP183 - N:UNK1 | 4.73478 | Hydrophobic | Pi-Alkyl |
|  | B:TRP183 - N:UNK1 | 4.65683 | Hydrophobic | Pi-Alkyl |
|  | B:TRP183 - N:UNK1 | 3.86334 | Hydrophobic | Pi-Alkyl |
|  | B:TRP183 - N:UNK1 | 4.84162 | Hydrophobic | Pi-Alkyl |

**Table S5.** Interactions of amino acids of the Glucose transporter 3 [GLUT 3] (PDB ID: 4ZWB) with the selected compounds, Lupeol (**C1**), β-sitosterol (**C2**), and 1-monostearin (**C3**) from *Justica gendarussa* during molecular docking.

| **compound** | **Bond (AA & Ligand)** | **Bond Length** | **Bond Type** | **Bond Nature** |
| --- | --- | --- | --- | --- |
| **C1** | A:VAL67 - N:UNK1 | 5.26817 | Hydrophobic | Alkyl |
|  | A:ILE166 - N:UNK1 | 5.1075 | Hydrophobic | Alkyl |
|  | A:ILE285 - N:UNK1 | 5.12099 | Hydrophobic | Alkyl |
|  | N:UNK1 - A:ILE162 | 5.06567 | Hydrophobic | Alkyl |
|  | N:UNK1:C - A:ILE162 | 5.4014 | Hydrophobic | Alkyl |
|  | N:UNK1:C - A:VAL163 | 4.77423 | Hydrophobic | Alkyl |
|  | N:UNK1:C - A:ILE166 | 4.5175 | Hydrophobic | Alkyl |
|  | N:UNK1:C - A:ILE285 | 4.02429 | Hydrophobic | Alkyl |
|  | A:PHE70 - N:UNK1:C | 5.43361 | Hydrophobic | Pi-Alkyl |
|  | A:PHE70 - N:UNK1:C | 4.8238 | Hydrophobic | Pi-Alkyl |
|  | A:PHE289 - N:UNK1 | 5.20718 | Hydrophobic | Pi-Alkyl |
|  | A:TYR290 - N:UNK1 | 5.4115 | Hydrophobic | Pi-Alkyl |
|  | A:TYR290 - N:UNK1:C | 4.5842 | Hydrophobic | Pi-Alkyl |
|  | A:PHE377 - N:UNK1:C | 3.73075 | Hydrophobic | Pi-Alkyl |
| **C2** | A:GLN159:HE21 - N:UNK1:O | 2.58758 | Hydrogen Bond | Conventional Hydrogen Bond |
|  | N:UNK1:C - A:TYR290 | 3.45191 | Hydrophobic | Pi-Sigma |
|  | A:ILE166 - N:UNK1 | 4.21665 | Hydrophobic | Alkyl |
|  | N:UNK1 - A:ILE166 | 5.41465 | Hydrophobic | Alkyl |
|  | N:UNK1 - A:ILE285 | 4.45372 | Hydrophobic | Alkyl |
|  | N:UNK1:C - A:ILE285 | 3.40132 | Hydrophobic | Alkyl |
|  | N:UNK1:C - A:VAL67 | 4.71238 | Hydrophobic | Alkyl |
|  | N:UNK1:C - A:VAL67 | 4.65429 | Hydrophobic | Alkyl |
|  | A:PHE289 - N:UNK1 | 4.13057 | Hydrophobic | Pi-Alkyl |
|  | A:PHE289 - N:UNK1 | 5.30383 | Hydrophobic | Pi-Alkyl |
|  | A:PHE289 - N:UNK1:C | 4.92294 | Hydrophobic | Pi-Alkyl |
| **C3** | A:ASN286:HD21 - N:UNK1:O | 2.18623 | Hydrogen Bond | Conventional Hydrogen Bond |
|  | N:UNK1:H - A:ASN413:OD1 | 2.12646 | Hydrogen Bond | Conventional Hydrogen Bond |
|  | N:UNK1:C - A:THR28:OG1 | 3.58812 | Hydrogen Bond | Carbon Hydrogen Bond |
|  | N:UNK1 - A:ILE166 | 4.69477 | Hydrophobic | Alkyl |
|  | N:UNK1 - A:ILE285 | 4.86517 | Hydrophobic | Alkyl |
|  | N:UNK1 - A:ILE162 | 5.46063 | Hydrophobic | Alkyl |
|  | N:UNK1:C - A:ILE285 | 5.02424 | Hydrophobic | Alkyl |
|  | A:PHE24 - N:UNK1 | 4.92114 | Hydrophobic | Pi-Alkyl |
|  | A:PHE289 - N:UNK1 | 5.42165 | Hydrophobic | Pi-Alkyl |
|  | A:PHE289 - N:UNK1 | 4.20976 | Hydrophobic | Pi-Alkyl |
|  | A:PHE377 - N:UNK1:C | 4.7507 | Hydrophobic | Pi-Alkyl |

**Table S6.** Interactions of amino acids of the dihydrofolate reductase (DHFR) (PDB ID: 4M6J) with the selected compounds, Lupeol (**C1**), β-sitosterol (**C2**), and 1-monostearin (**C3**) from *Justica gendarussa* during molecular docking.

| **Compound** | **Bond (AA & Ligand)** | **Bond Length** | **Bond Type** | **Bond Nature** |
| --- | --- | --- | --- | --- |
| **C1** | N:UNK1:H - A:ILE16:O | 2.82999 | Hydrogen Bond | Conventional Hydrogen Bond |
|  | A:GLY17:CA - N:UNK1:O | 3.45921 | Hydrogen Bond | Carbon Hydrogen Bond |
|  | A:LYS55 - N:UNK1 | 5.34513 | Hydrophobic | Alkyl |
|  | A:LYS55 - N:UNK1 | 5.06917 | Hydrophobic | Alkyl |
|  | N:UNK1 - A:ILE16 | 5.14934 | Hydrophobic | Alkyl |
|  | N:UNK1:C - A:LYS55 | 5.41745 | Hydrophobic | Alkyl |
| **C2** | A:ILE16 - N:UNK1 | 4.82178 | Hydrophobic | Alkyl |
|  | N:UNK1 - A:LEU22 | 4.94352 | Hydrophobic | Alkyl |
|  | N:UNK1:C - A:LYS55 | 3.88935 | Hydrophobic | Alkyl |
|  | N:UNK1:C - A:LYS55 | 4.38619 | Hydrophobic | Alkyl |
| **C3** | A:ALA9:HN - N:UNK1:O | 2.47577 | Hydrogen Bond | Conventional Hydrogen Bond |
|  | N:UNK1:H - N:UNK1:O | 2.85277 | Hydrogen Bond | Conventional Hydrogen Bond |
|  | A:VAL8:CA - N:UNK1:O | 3.19788 | Hydrogen Bond | Carbon Hydrogen Bond |
|  | A:LYS55 - N:UNK1 | 5.35292 | Hydrophobic | Alkyl |
|  | A:LYS55 - N:UNK1 | 4.80461 | Hydrophobic | Alkyl |
|  | N:UNK1 - A:ILE16 | 4.2037 | Hydrophobic | Alkyl |
|  | A:TYR121 - N:UNK1 | 5.45154 | Hydrophobic | Pi-Alkyl |
